# Novel Antimicrobial Activities of Albofungin, Albonoursin, and Ribonucleosides Produced by *Streptomyces* sp. Caat 5-35 Against Phytopathogens and Their Potential as a Biocontrol Agent

**DOI:** 10.3390/molecules31010021

**Published:** 2025-12-20

**Authors:** Carmen Julia Pedroza-Padilla, Sergio Orduz, Danilo Tosta Souza, Geraldo Antonio Astolpho-Barbão, Luiz Alberto Beraldo Moraes

**Affiliations:** 1 Grupo BiotecGen, Departamento de Biología, Microbiología y Afines, Facultad de Ciencias Básicas, Universidad Popular del Cesar, Diagonal 21 # 29-56, Valledupar 200004, Colombia; 2 Departamento de Biociencias, Facultad de Ciencias, Universidad Nacional de Colombia, Sede Medellín, Carrera 65 # 59A-110, Medellín 050034, Colombia; 3 Departamento de Química, Faculdade de Filosofia, Ciências e Letras de Ribeirão Preto—FFCLRP, Universidade de São Paulo-USP, Ribeirão Preto 14040-901, SP, Brazil; danilo_tosta@hotmail.com (D.T.S.); geraldoaab11@hotmail.com (G.A.A.-B.); 4 Embrapa Meio Ambiente, Jaguariúna 13918-110, SP, Brazil

**Keywords:** albofungin, albonoursin, phytopathogenic microorganisms, 2-chloroadenosine, natural products, antimicrobial, *Theobroma cacao*, biocontrol

## Abstract

The genus *Streptomyces* is the largest group within the phylum Actinobacteria, recognized for producing antibiotics and enzymes, with wide applications in medicine and biological control for crop protection against phytopathogens. In this study, the *Streptomyces* sp. Caat 5-35 strain, isolated from soil of the Caatinga biome in Brazil, and identified by analysis of the 16S rRNA gene, demonstrated its antagonistic effect *in vitro* in dual cultures against *Phytophthora palmivora*, *Colletotrichum acutatum*, *Fusarium oxysporum*, *Rhizoctonia solani*, *Sclerotinia sclerotiorum*, and *Fusarium graminearum*. Caat 5-35 inhibited mycelial growth ranging from 19% to 73.3%. Compounds purified by prep-HPLC from extracts were identified by spectral data analysis using UHPLC-triple-TOF-MS/MS, or nuclear magnetic resonance (NMR). This work demonstrated for the first time the anti-oomycete activity of albofungin, its derivatives, and albonoursin against *P. palmivora*. Moreover, the growth inhibition of *Colletotrichum gloeosporioides* by albonoursin and the antibacterial effect of 2-chloroadenosine and 5′-O-sulfamoyl-2-chloroadenosine against *Pectobacterium carotovorum* were demonstrated as novel findings. Caat 5-35 exhibited the ability to solubilize phosphates and produce cellulases on CMC agar. The findings of this study, in combination with *in vitro* bioassays on cacao pods (*Theobroma cacao* L.) inoculated with the antagonist strain and *P. palmivora* APB-35, demonstrate that *Streptomyces* sp. Caat 5-35 is a source of natural products with applications in agriculture and could serve as an alternative for crop protection.

## 1. Introduction

Cacao (*Theobroma cacao* L.) and oil palm (*Elaeis guineensis* Jacq.) are crops in high demand worldwide, with a significant economic impact on developing countries such as Colombia. These agricultural systems report losses at around 23% due to the phytosanitary effects of pests [1]. The oomycete *Phytophthora palmivora* (Butler) is the causal agent of various phytosanitary diseases, including Black Pod Rot (BPR) in cacao [2] and Bud Rot (BR) in oil palm [3,4] and to mitigate the negative impact, these microorganisms are controlled by adjusting drainage systems, pruning trees, removing weeds, stripping diseased fruit, and applying systemic or contact fungicides [1]. Then, microorganisms emerge as a strategy to promote plant growth and reduce the use of chemical pesticides [5,6]. The genus *Streptomyces* is well known for producing structurally diverse secondary metabolites and lytic enzymes with antimicrobial activity; therefore, they are an alternative for disease control in crops [7,8,9,10].

Among the antimicrobials produced by this group of microorganisms is albofungin (also known as kanchanomycin), a polycyclic xanthone with a hydrazine modification synthesized by type II polyketide synthases (PKSs). Albofungin is produced by some actinomycete species that have shown bioactivity against Gram-negative and Gram-positive pathogenic bacteria, yeasts, filamentous fungi, and tumour cell lines [11,12,13]. Another metabolite produced by *Streptomyces* sp. is albonoursin [cycle (ΔPhe-ΔLeu)]. This cyclic dipeptide belongs to the 2.5-diketopiperazine (DKP) family with antibacterial activity obtained from *Streptomyces albus* and *Streptomyces noursei* [14,15]. Albonoursin synthesis is catalyzed by cyclodipeptide synthases (CDPS or AlbC protein) with dehydrogenations introduced by the cyclopeptide oxidase enzyme (or cyclic dipeptide oxidase, CDO) to the natural substrate cyclo(L-Phe-L-Leu) [16,17]. The proteins involved in albonoursin biosynthesis are encoded by the *albA*, *albB*, and *albC* genes [16]. Other metabolites are 2-chloroadenosine and 5′-O-sulfamoyl-2-chloroadenosine (dealanylascamycin, DACM), which are ribonucleoside antibiotics of microbial origin with structural modifications (addition of a chlorine atom at position two and a sulfamoyl group at carbon five instead of a phosphate group), which enhance their antibacterial activity [18,19,20]. Recent studies have demonstrated the antimalarial activity of DACM, with potent inhibition of *Plasmodium falciparum* growth [21].

This study identified the metabolites responsible for the bioactivity of *Streptomyces* sp. Caat 5-35 against various phytopathogens and evaluated its potential as a biocontrol agent. Initially, the antimicrobial compounds produced in the ISP2 medium were extracted. Subsequently, the compounds active against the evaluated pathogens were purified and identified. Furthermore, the cellulases production and the ability to solubilize phosphates were detected, and the *in vitro* biocontrol effect of Caat 5-35 against *P. palmivora* APB-35 in cacao fruits was verified. To our knowledge, this is the first study to demonstrate the anti-oomycete effect of albofungin, its derivatives, and albonoursin against *P. palmivora*, the antifungal activity of albonoursin against *Colletotrichum gloeosporioides*, and its antibacterial effect against *Pectobacterium carotovorum* by the compounds 2-chloroadenosine and 5′-O-sulfamoyl-2-chloroadenosine.

## 2. Results

### 2.1. Molecular Identification and In Vitro Antagonism Assay of Isolate Caat 5-35

**Figure 1:**
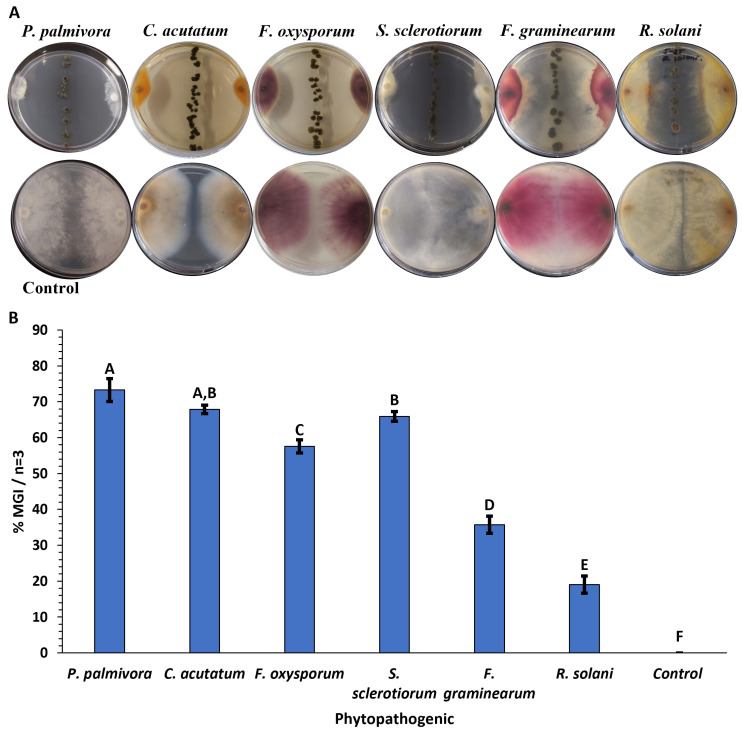
Antagonistic effect of *Streptomyces* sp. Caat 5-35 against phytopathogenic microorganisms. (**A**) Dual culture assays on PDA and carrot agar; the control was a monoculture of the pathogen. (**B**) Inhibition percentage of mycelial growth (%MGI) of phytopathogens by Caat 5-35. The bars represent the standard error. Means (n = 3) that do not share a letter are significantly different according to Tukey’s test (α = 0.05).

The isolate from the soil of Cactaceae in the Caatinga biome in Brazil showed identity with *Streptomyces paromomycinus* NBRC 15454 (99.86%) and *Streptomyces chrestomyceticus* NBRC 13444 (99.79%). Phylogenetic trees constructed from the 16S rDNA nucleotide sequence (1459 bp) suggest that Caat 5-35 is closely related to both species (Figures S1 and S2); as a result, the isolate was identified as *Streptomyces* sp. Caat 5-35. The antagonistic effect of *Streptomyces* sp. Caat 5-35 against the oomycete and the five fungi evaluated ranged from 19% to 73% of MGI compared to the control (Figure 1). Dual tests against *P. palmivora* APB-35, *C. acutatum,* and *S. sclerotiorum* showed the highest mycelial growth inhibition (73.3%, 67.9%, and 65.9%, respectively). The lowest antifungal effect was observed against *R. solani*.

### 2.2. Purification and UPLC-MS Analysis of Antimicrobial Compounds Produced by Streptomyces *sp.* Caat 5-35

**Figure 2:**
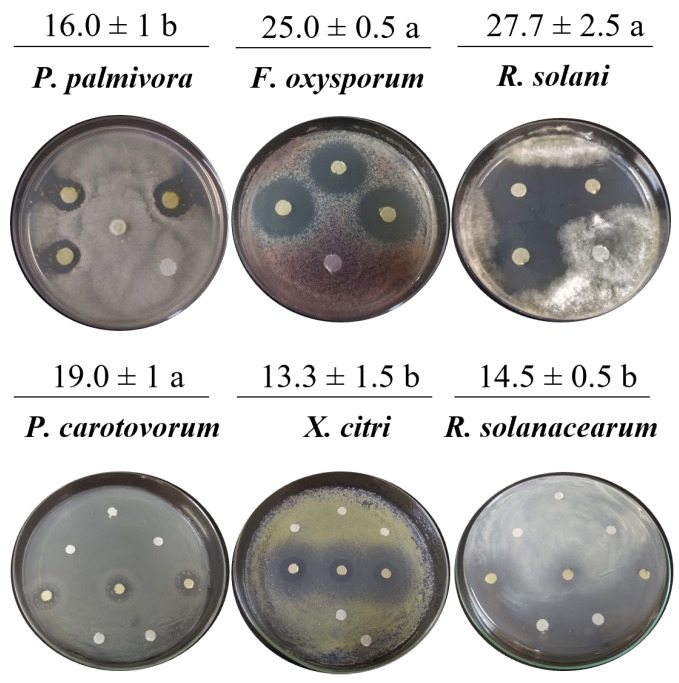
Inhibition halo produced by the extract of *Streptomyces* sp. Caat 5-35 against the phytopathogenic strain. The intervals represent the standard error of the mean (n = 3). Mean values that do not share the same letter are significantly different according to Tukey’s test (α = 0.05). The inhibition zone is shown in millimetres (mm).

Antimicrobial evaluation of the dry residue resuspended in methanol or DMSO showed anti-oomycete activity against *P. palmivora* APB-35, with mycelial growth inhibition halos of 16 mm. The methanol and DMSO controls showed no antimicrobial effect. Additionally, the extract exhibited antifungal activity against *F. oxysporum*, *R. solani*, and *C. gloeosporioides* (inhibition halo of 27.0 ± 1 mm), as well as antibacterial activity against *P. carotovorum*, *X. citri*, and *R. solanacearum* (Figure 2). Significant differences were found in all tests compared to the negative control. No differences in inhibition halo size were observed among the fungi evaluated. A greater antibacterial effect was found against *P. carotovorum*. The extract obtained from the uninoculated ISP2 medium did not inhibit the growth of phytopathogenic microorganisms, nor did it exhibit ions associated with the antimicrobial compounds in this work.

The LC-UV chromatogram exhibited two groups of predominant peaks at 325 and 370 nm. The first, between 6 and 7 min, was eluted with 40% acetonitrile and 0.1% formic acid, and the second one, between 10 and 14 min, was eluted with 50–60% of the same phase B. The dried residues obtained from the 50 collected fractions were then evaluated against *P. palmivora* APB-35 and *P. carotovorum*. Table 1 summarizes the results of the mycelial growth inhibition halo by the disc diffusion method in carrot agar of the active fractions against *P. palmivora* APB-35. Of the 50 fractions collected, 10 showed an anti-oomycete effect, with significant differences compared to the negative control. To gain insight into the compounds, both the extract and the antimicrobial fractions were analyzed by UPLC-ESI-MS in positive and negative modes.

UPLC-MS analysis of the *Streptomyces* sp. Caat 5-35 extract revealed peaks with different retention times, some of which shared the same UV absorption pattern. The masses corresponding to the [M+H]+ ions of *m*/*z* 257 and 324 showed the highest absorption at 315 nm; [M+H]+ of *m*/*z* 506, 507, 520 and 521 at 250 and 373 nm; and finally, [M+H]+ of *m*/*z* 291 with the highest absorption at 337 nm, 555 (302 and 380 nm) and 478 (319 and 352 nm), respectively. Based on the UV absorption pattern, we hypothesized that some of the detected compounds could belong to a similar chemical class. In this study, the fractions that showed anti-oomycete activity were evaluated against *C. gloeosporioides*, and their antifungal activity was confirmed. The inhibition of *C. gloeosporioides* by the extract and fractions 12, 13, and 14 did not show significant differences compared with the positive control, Difenoconazole (FUNGIWAY^®^) (Table 1).

**Figure 3:**
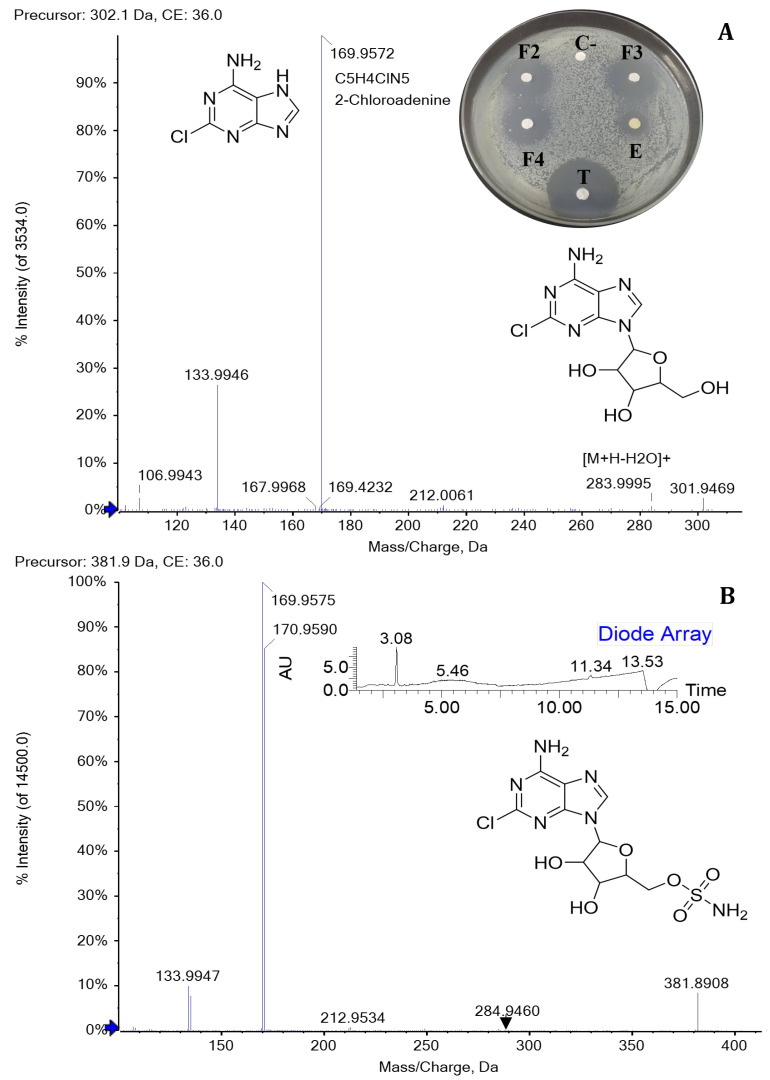
UHPLC-TOF-MS/MS spectra of 2-chloroadenosine and 5′-O-sulfamoyl-2-chloroadenosine precursors produced by *Streptomyces* sp. Caat 5-35. (**A**) MS/MS fragmentation of [M+H]^+^ of *m*/*z* 302.06, highlighting the chemical structures and bioassays with inhibition halos for fractions (F2, F3, and F4) and the crude extract against *P. carotovorum*. (**B**) MS/MS fragmentation of [M+H]^+^ of *m*/*z* 381.03, with the structure and DAD chromatogram of fraction 3. E: extract, C-: negative control, T: tetracycline.

For the bioassays of the 50 fractions against *P. carotovorum*, only inhibition halos were observed for fractions 2, 3, and 4 (eluted between 30 and 35% ACN with UV absorption at 261 nm). The three fractions showed significant differences compared to the negative control. The crude extract exhibited lower antimicrobial activity than the fractions, and all treatments inhibited less than tetracycline (Figure 3A, Table 2). Furthermore, an intense signal was found among the peaks detected in F8 during UPLC-MS analysis. This signal corresponds with the [M+H]+ ion of *m*/*z* 259.1441, which was associated with cyclo (ΔPhe-L-Leu) or ΔcFL, an intermediate in the albonoursin biosynthesis that has a dehydrogenation catalyzed by the CDO enzyme [22].

### 2.3. Identification of Antimicrobial Compounds by UHPLC-TOF-MS/MS and NMR

To identify the metabolites responsible for antibacterial activity against *P. carotovorum*, UHPLC-TOF-MS analysis was performed on active fractions 2, 3, and 4. The results showed the presence of the molecular ion [M+H]+ of *m*/*z* 302.0652 and 381.0384, the isotopic pattern due to the presence of chlorine for both molecules, 304.0622 and 383.0356 with 1/3 intensity, and the respective sodium adducts [M+Na]^+^ of *m*/*z* 324.1494 and 403.0198 Da, with UV absorption at 261 nm. The MS/MS fragmentation spectrum of the precursor [M+H]^+^ of *m*/*z* 302.06 showed the ionic fragment *m*/*z* 170 typical of 2-chloroadenine, which was generated by the loss of ribose. In addition, the fragmentation pattern of the MS/MS analysis of the precursor [M+H]^+^ of *m*/*z* 381.03, when magnifying the spectrum image, showed ions formed by the loss of water [M+H-H_2_O]^+^ of *m*/*z* 363.2985, the sulfamoyl group [M-H_2_NO_2_S-H_2_O+H]^+^ of *m*/*z* 284.9460, and 2-chloroadenine [M-C_5_H_4_ClN_5_+H]^+^ of *m*/*z* 212.9534 (Figure 3). Therefore, the compounds inhibiting the growth of *P. carotovorum* belong to the same chemical family and are consistent with the identification of 2-chloroadenosine and 5′-O-sulfamoyl-2-chloroadenosine described in the literature [20,23].

**Figure 4:**
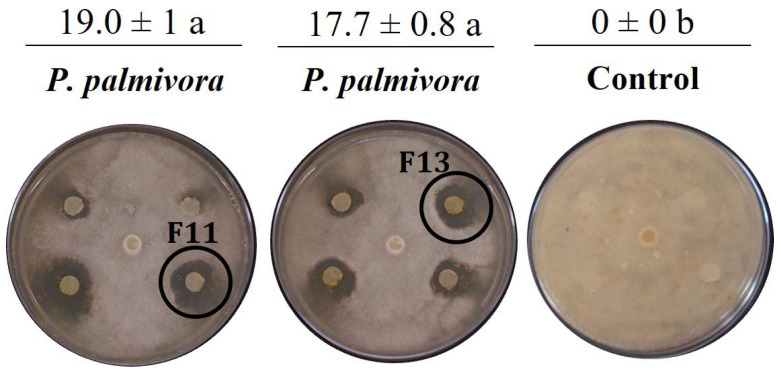
Inhibition of mycelial growth of *P. palmivora* APB-35 by albonoursin and albofungin purified from *Streptomyces* Caat 5-35. The intervals represent the standard error of the mean (n = 3). Mean values that do not share the same letter are significantly different according to Tukey’s test (α = 0.05). The inhibition zone is shown in millimetres (mm). Disk size: 5.0 mm.

**Figure 5:**
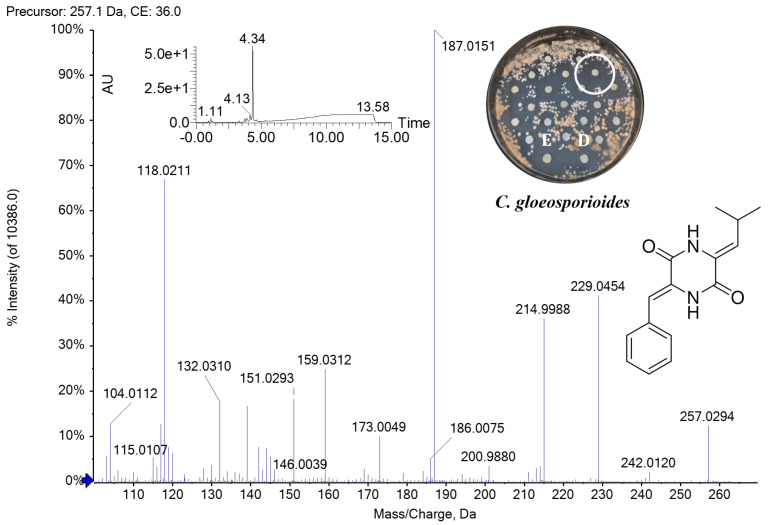
UHPLC-TOF-MS/MS spectrum of albonoursin produced by *Streptomyces* sp. Caat 5-35. Precursor [M+H]^+^ of *m*/*z* 257.13. The DAD chromatogram, chemical structure, and bioassay of the antimicrobial activity of albonoursin (circle, F11), against *C. gloeosporioides* are shown. E: extract. D: difenoconazole (FUNGIWAY^®^).

High-resolution mass spectrometry (HRMS) fragmentation analyses of fraction 11 with anti-oomycete activity against *P. palmivora* APB-35 and antifungal activity against *C. gloeosporioides* confirmed that *Streptomyces* sp. Caat 5-35 produces DKP known as albonoursin (Figure 4, Table 2), a cyclic dipeptide of phenylalanine and leucine [cycle (ΔPhe-ΔLeu)]. This showed the highest UV absorption at 315 nm in the DAD chromatogram. The HRMS spectrum showed ions of *m*/*z* 257.1393, 279.1110, and 513.2513, corresponding to species [M+H]^+^, [M+Na]^+^, and [2M+H]^+^, respectively. MS/MS fragmentation (Figure 5), obtained from the precursor [M+H]^+^ of *m*/*z* 257.13, revealed the loss of carbon monoxide [M+H-CO]^+^ of *m*/*z* 229.04 and the characteristic ionic fragments (*m*/*z* 215, 187, 201, and 146) described in other studies for albonoursin [24]. In fraction 12, several peaks with different absorptions were observed in the UV region. The high-intensity signal at 338 nm showed the protonated ion [M+H]^+^ of *m*/*z* 291.1132, the sodium adduct [M+Na]^+^ of *m*/*z* 313.0957, and the dimer [2M+H]^+^ of *m*/*z* 581.2189. These data allowed us to infer the assignment of piperafizine B.

HRMS analyses of the ions present in the fractions with anti-oomycete activity against *P. palmivora* APB-35 (Table 3) indicate that the active compounds are associated with albofungin derivatives (Figures S3–S5). From the experimental masses found, it can be inferred that the ions [M+H]^+^ of *m*/*z* 507.1413 (albofungin A) and *m*/*z* 506.1445 (chrestoxanthone A) correspond to the loss of a methyl and amino group, respectively, in albofungin. On the other hand, the *m*/*z* 520.1621 ion corresponds to the replacement of the amino group by a methyl group in the only remaining nitrogen atom in the molecule (albofungin B). Finally, the *m*/*z* 555.1169 ion (chloroalbofungin) corresponds to the addition of a chlorine atom to the chemical structure of albofungin [M+H]^+^ of *m*/*z* 521.1568 (Figure S6). These observations are consistent with the results reported in previous studies [11,25,26].

To demonstrate that albofungin is one of the compounds responsible for anti-oomycete activity, fraction 13, with deuterated chloroform, was subjected to 1D and 2D nuclear magnetic resonance analysis (NMR), obtaining the 1H NMR spectrum, gHMQC (Heteronuclear Multiple Quantum Coherence), and gHMBC (Heteronuclear Multiple Bond Coherence) (Figures S7 and S8). The spectroscopic data presented in Table S1 are consistent with previous reports [12,13]. To confirm the structure of albofungin, correlations between hydrogen and carbon were observed. The δH 6.86 ppm signal was attributed to hydrogen H6 and showed a long-distance correlation with carbons with δC 37.0 (C-8); δC 113.9 (C-6); δC 110.3 (C-5), and δC 106.0 (C-4). In the context of the research, an association was observed between the δH 4.24 ppm signal and H-21, suggesting a significant long-distance correlation with the carbons δC 120.5 (C-25); δC 164.2 (C-20); δC 25.1 (C-22), and δC 58.8 (C-28). The present study confirmed that *Streptomyces* sp. Caat 5-35 produces the polycyclic xanthone albofungin, which inhibits the mycelial growth of *P. palmivora* APB-35.

### 2.4. Lytic Enzymes, Phosphate Solubilization, and in Vitro Assays in Cacao

**Figure 6:**
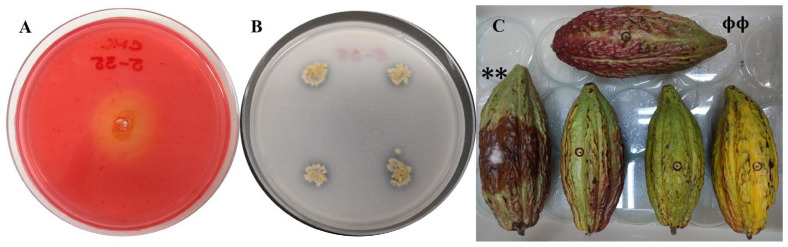
Cellulolytic activity, phosphate solubilization, and *in vitro* biocontrol in cacao by *Streptomyces* sp. Caat 5-35. (**A**) Degradation halo in CMC agar. (**B**) Phosphate solubilization halo in NBRIP medium. (**C**) Biocontrol effect in cacao fruits inoculated with *P. palmivora* APB-35 and treated with Caat 5-35. (**): pod treated with sterile water and oomycete. (ϕϕ): pod treated with sterile water in the absence of the pathogen. Treatment: pods treated with Caat 5-35, and agar plug with *P. palmivora* APB-35. Lesion area on cacao pod control **: 100.5 ± 25.9 cm^2^. The intervals represent the standard error of the mean (n = 10).

Cellulolytic activity assays in CMC agar were revealed with Congo Red. The results showed a 25 ± 1.0 mm degradation halo around the well, indicating that *Streptomyces* sp. Caat 5-35 produces cellulases capable of hydrolyzing the cellulose present in the medium. This lytic activity suggests a possible mechanism involved in its antagonistic action against *P. palmivora* APB-35. Additionally, the ability of Caat 5-35 to solubilize tricalcium phosphate in NBRIP medium was demonstrated by the presence of clear halos around the colony, with a PSI of 1.5 ± 0.1 (Figure 6A,B). This trait could indirectly contribute to controlling the phytopathogen, as greater phosphorus availability can promote the host plant’s health and vigour, making it less susceptible to pathogen attack.

The cacao fruits or pods treated with a Cat 5-35 suspension and inoculated with a plug of *P. palmivora* APB-35 showed no evidence of lesions, consistent with the control treated with sterile distilled water, which did not contain the phytopathogen. In contrast, pods treated with sterile distilled water and agar plugs containing oomycete developed extensive dark brown necrotic lesions, which also showed evidence of aerial mycelial growth of *P. palmivora* APB-35 (Figure 6C).

## 3. Discussion

The *Streptomyces* species are Actinobacteria that produce bioactive natural substances, which are recognized for their capacity to synthesize antibiotics [6,27]. In this study, we demonstrated that the antagonist bacterium *Streptomyces* sp. Caat 5-35 exhibits broad-spectrum growth inhibition of phytopathogens, including one oomycete and four fungal genera, spanning two taxonomic divisions (Ascomycota and Basidiomycota). The antimicrobial activity of the Caat 5-35 was evaluated by observing the secretion of diffusible metabolites in the culture medium, which inhibited the *in vitro* mycelial growth of *P. palmivora*, *C. acutatum*, *F. oxysporum*, *R. solani*, *S. sclerotiorum*, and *F. graminearum*. In the literature, the anti-oomycete effect of *Streptomyces* sp. in dual cultures against *P. palmivora* has been described, as well as its potential as a bioinoculant and plant growth promoter in crops [28]. The findings of this research on antagonism against the oomycete in question are consistent with the 75.5% reduction in growth of *P. palmivora* caused by *Streptomyces plicatus* B4-7 [29] and the 77.3% reduction caused by *B. velezensis* FZB42 [30]. In the present study, the MGI of *C. acutatum* and *F. oxysporum* by Caat 5-35 was higher than that reported by Kim et al. (2024) for *Streptomyces* sp. B-1662 against these fungi (52% and 38.4%, respectively) [31]. In dual assays, *Streptomyces roietensis* TCS21-117 demonstrated its ability to inhibit the growth of several pathogenic species, including *R. solani*, *F. graminearum*, *P. capsici*, *S. sclerotiorum*, and *F. oxysporum* [27]. In addition, multiple studies have corroborated the antifungal effects of different *Streptomyces* species against *F. oxysporum* [32], *R. solani*, *S. sclerotiorum*, *F. graminearum* [33], *C. acutatum*, and *C. gloeosporioides* [34].

This study demonstrated that the antagonistic bacterium *Streptomyces* sp. Caat 5-35 produces albofungin and its derivatives with anti-oomycete effect against the phytopathogen *P. palmivora* APB-35. The antifungal activity reported in this work for the polycyclic xanthone is consistent with the inhibition of *C. gloeosporioides* growth by the antibiotics albofungin, chrestoxanthone A, chrestoxanthone C, and chloroalbofungin produced by *S. chrestomyceticus* BCC 24770 in LS2 medium [25]. While the extract demonstrated activity against *C. acutatum*, *F. oxysporum*, *R. solani*, *S. sclerotiorum*, and *F. graminearum*, the compounds responsible for the observed antifungal activity remain to be identified for these pathogens. The mechanism of action of albofungin and its derivatives in inhibiting the mycelial growth of oomycetes and phytopathogenic fungi remains unknown; however, reports describe their action against antibiotic-resistant bacteria. These indicate that albofungin binding to bacterial transglycosylase (TGase) directly affects cell wall biosynthesis [13]. TGase is a protein that catalyzes the polymerization of peptidoglycan glucan chains during bacterial wall synthesis [35]. In addition, in *Vibrio parahaemolyticus*, albofungin alters permeabilization of the cytoplasmic membrane by forming pores that facilitate the entry of the antimicrobial into the cell. This compound then inhibits peptidoglycan synthesis, interacts with DNA, and affects enzymes involved in the replication process [36].

The MS/MS fragmentation spectrum of albonoursin (c(ΔPΔL) or ΔΔcFL) obtained from the analysis of fraction 11 is consistent with the antibacterial cyclic dipeptide produced by *Nocardiopsis alba* [24] and *Streptomyces noursei* [37]. The present study demonstrates the anti-oomycete activity of albonoursin against *P. palmivora* APB-35 and the antifungal activity against *C. gloeosporioides*, information previously unknown. The specific mode of action of this compound on these pathogens has not yet been clarified. However, previous studies have described the inhibitory effect of the DKP cycle (L-Pro-L-Tyr) produced by *Lysobacter capsici* AZ78 on the development of sporangia of *Phytophthora infestans* and *Plasmopara viticola* [38]. Furthermore, the AZ78 strain has been observed to affect mycelial growth in antagonism tests of four species of *Phytophthora* and *Pythium ultimum* [38]. Additionally, transmission electron microscopy analysis showed that three types of DKPs produced by the endophytic fungus *Alternaria alternata* deformed the cell wall, condensed the cytoplasm, caused abnormal vacuolization, and necrosed the haustoria of *P. viticola*, however, without causing damage to the vine tissue [39]. Recently, it has been reported that DKP produced by *Bacillus subtilis* KS1, cycle(L-Leu-L-Phe), a precursor of albonoursin, inhibits haustorium formation, but not the germination of zoospores of the obligate parasite *P. viticola*, suggesting a possible involvement of DKP with recognition receptors and disruption of the plasma membrane [40].

The identification of 2-chloroadenosine and 5′-O-sulfamoyl-2-chloroadenosine as the antibacterial compounds active against *P. carotovorum* was supported by their fragmentation patterns, which matched those described in earlier studies [20,23]. Both antibiotics produced by *Streptomyces* strains exhibit antibacterial activity against Gram-positive and Gram-negative bacteria [18,19]. In an effort to elucidate the mode of action of 5′-O-sulfamoyl-2-chloroadenosine against *X. citri* and *Xanthomonas oryzae*, it was demonstrated that this antibiotic is more effective against Gram-negative bacteria and acts through cell membrane permeation and inhibition of protein synthesis in *X. citri* [18]. Recently, the antimalarial activity of DACM produced by *Streptomyces* sp. was reported, in which the sulfamate nucleoside mimics adenosine monophosphate (AMP) to block aminoacyl-tRNA synthetase activity and inhibit protein translation in the *P. falciparum* parasite [21].

In this study, no antibacterial activity was observed in fractions containing albofungin and its derivatives, or in the DKPs, against *P. carotovorum*. Therefore, we infer that the inhibition of the growth of *X. citri* and *R. solanacearum* EAP05 could be related to the 2-chloroadenosine and 5′-O-sulfamoyl-2-chloroadenosine present in the extract obtained from the culture of *Streptomyces* sp. Caat 5-35 in ISP2. This hypothesis is consistent with previous research [18]. Moreover, earlier studies reported no inhibition zones in antibacterial activity assays against *Erwinia carotovora* subsp. *carotovora*, *R. solanacearum*, and *Xanthomonas campestris* by the secondary metabolites produced by *Lysobacter capsici* AZ78 [41,42]. Although this strain secreted albonoursin and other DKPs, it did not affect the growth of these phytopathogenic bacteria [41,42]. As far as we know, this finding provides the first evidence of the antibacterial activity of 2-chloroadenosine and 5′-O-sulfamoyl-2-chloroadenosine against *P. carotovorum*. Moreover, it suggests their potential biological role in inhibiting the growth of *X. citri* and *R. solanacearum*.

The absence of lesions in cacao fruits treated with *Streptomyces* sp. Caat 5-35 demonstrated the strong biocontrol activity of the antagonistic bacterium against *P. palmivora* under *in vitro* conditions. While the production of antimicrobial compounds is a probable mechanism in the protective effect of cacao pods, the *in vitro* experiment does not rule out the contribution of other direct or indirect mechanisms, such as induced resistance, lytic enzymes, or competition. Previous reports of bioassays on cacao pods inoculated with a strain of *Pseudomonas* sp. 1 that produces surfactants showed inhibition of symptoms caused by *P. palmivora* in detached fruits [43]. Similar results were also observed against the same oomycete with *P. aeruginosa* and *Chryseobacterium proteolyticum*, both bacteria that produce volatile compounds and lytic enzymes (cellulases, lipases, proteases, and pectinases) [44]. The detection of extracellular lytic cellulase-type enzymes produced by *Streptomyces* sp. Caat 5-35 is relevant. This is because the cell walls of oomycetes, unlike those of fungi, contain cellulose, glucans, and, to a lesser extent, chitin [45,46]. Our hypothesis is consistent with the inhibition of *P. palmivora* mycelial growth by proteins purified from the supernatant of *P. aeruginosa* RS1 culture [47].

The phosphate-solubilizing capacity of *Streptomyces* sp. Caat 5-35, observed in NBRIP medium, supports its potential role as a plant growth–promoting microorganism. This mechanism, which enhances the bioavailability of previously insoluble phosphate forms, has also been described in several organisms, including *Bacillus* sp. and *Streptomyces* sp. [28,48]. However, further experiments are needed to elucidate the mechanism of action of the antimicrobial compounds produced by Caat 5-35, including genome sequencing and analysis. Furthermore, identify the compounds responsible for antifungal activity against *C. acutatum*, *F. oxysporum*, *R. solani*, *S. sclerotiorum*, and *F. graminearum*; evaluate the biocontrol effect through greenhouse and field trials; quantify the antimicrobial activity of metabolites; and promote plant growth by Actinobacteria. The results of this work demonstrate that *Streptomyces* sp. Caat 5-35 is an antagonistic bacterium with anti-oomycete, antifungal, and antibacterial effects, capable of producing structurally diverse antimicrobial compounds that directly inhibit the growth of *P. palmivora*, *C. gloeosporioides*, *P. carotovorum*, and other phytopathogens. Consequently, Caat 5-35 could be an alternative for the biological control of crop diseases.

## 4. Materials and Methods

### 4.1. Phytopathogenic Microorganisms and Growing Conditions

Caat 5-35 was isolated from soil in the Caatinga biome in Jutaí, Pernambuco, Brazil (08°36′13.17″ S; 40°13′4.18″ W), in collaboration with the Environmental Microbiology Laboratory at EMBRAPA Environment, coordinated by Dr Itamar Soares de Melo. The strain was deposited in the EMBRAPA microorganism collection (Coleção de Microrganismos de Importância Agrícola e Ambiental, CMAA, Jaguariúna, São Paulo, Brazil) and reactivated in ISP2 agar or potato dextrose agar (PDA, Oxoid, Basingstoke, UK). The oomycete *P. palmivora* APB-35 was provided by Dr Lucía Afanador Kafuri, professor at the Universidad Nacional de Colombia, Medellín campus. For the reactivation of APB-35, an agar plug with grown mycelium preserved in sterile water at room temperature was placed on carrot agar in a Petri dish (AZ, consisting of 200 g/L carrot, 15 g/L agar, and distilled water, with pH adjusted to 7.0 ± 0.2), and incubated at 30 °C for 7 days. *Fusarium oxysporum* 307 (FOC-307) was provided by Dr. Rafael Eduardo Arango Isaza, professor at the Universidad Nacional de Colombia, Medellín. *C. gloeosporioides* was donated by Dr David Granada García, researcher at the Corporación para Investigaciones Biológicas (Medellín, Colombia). *Rhizoctonia solani*, *Sclerotinia sclerotiorum*, *Fusarium graminearum*, *Colletotrichum acutatum*, *Fusarium oxysporum*, and *Xanthomonas citri* subsp. *citri* (*Xcc*) belongs to the phytopathogen collection of the LabEMass Laboratory at the Universidade de São Paulo (Ribeirão Preto, Brazil). The reactivation and subsequent testing of the fungi were carried out in PDA. The phytopathogenic bacteria *P. carotovorum* and *Ralstonia solanacearum* EAP05 were supplied by Professors Adriana González Almario (Universidad Nacional de Colombia, Bogotá) and Camilo Ramírez (Universidad de Antioquia), respectively. The bacterial strains were reactivated and cultured in broth and in modified Kelman’s agar (KMB and KMA, g/L: 5 glucose, 10 tryptone, 1 of yeast extract; only the solid medium contained agar; Oxoid, Basingstoke, UK).

### 4.2. Molecular Identification of Bacteria Isolated Caat 5-35

Molecular identification of strain Caat 5-35 was performed by extracting genomic DNA using the PureLink Genomic DNA kit (Invitrogen, Waltham, MA, USA) according to the manufacturer’s instructions. Amplification of the gene encoding 16S rRNA was performed by PCR with universal primers 27F (5′-AGAGTTTGATCMTGGCTCAG-3′) and 1492R (5′-GGTTACCTTGTTACGACTT-3′) as described in the literature [49]. The sequence obtained was compared with sequences deposited in the GenBank database (https://www.ncbi.nlm.nih.gov/genbank/, submitted on 20 September 2025) and on the EzTaxon server [50]. For phylogenetic analysis, reference sequences with high nucleotide identity (>97%) were selected and aligned using ClustalW in MEGA 11 (11th version, Philadelphia, PA, USA) [51]. Phylogenetic trees were inferred using the Maximum Likelihood (ML) [52] and Neighbour-Joining (NJ) algorithms [53]. The topology was evaluated using a Bootstrap analysis with 1000 repetitions. The most appropriate nucleotide substitution model was determined using MEGA’s own tools, selecting the Tamura 3 model, parameters (T92) for ML analysis, and the T92+G+I model for NJ tree construction. The 16S rDNA sequence of *Streptomyces albus* subsp. *albus* DSM 40313 was used as the outgroup, and the 16S rDNA sequence of strain Caat 5-35 was deposited in the GenBank database under accession number PX368896.

### 4.3. In Vitro Antagonism Assays

The antagonistic effect of strain Caat 5-35 against *P. palmivora* APB-35, *F. oxysporum*, *R. solani*, *S. sclerotiorum*, *F. graminearum*, and *C. acutatum* was evaluated by dual culture assays. For the oomycete, 30% (*w*/*v*) PDA was used. For the fungi, PDA was prepared according to the manufacturer’s instructions (Oxoid, Basingstoke, UK). In Petri dishes with solid medium, a five mm diameter agar plug with pathogen mycelium (from a 7-day culture) was placed at each end; then, a straight line was drawn in the centre of the dish from two colonies of isolate Caat 5-35 and incubated at 28 °C in darkness. The pathogen grown in the absence of the actinomycete served as the control. When the control mycelium spread to the centre of the plate, the growth radius (cm) was measured, and finally the percentage of mycelial growth inhibition was determined as follows: %MGI = [(C − T)/C] × 100 [44,54]. Where T is the mycelial growth radius in the presence of the bacterium, and C is the mycelial growth radius of the absolute control (without bacteria). The tests were performed in triplicate.

### 4.4. Production of Bioactives, Analysis, and Purification from the Extract

#### 4.4.1. Production of Antimicrobial Compounds by *Streptomyces* sp. Caat 5-35

From a three-day Caat 5-35 culture in ISP2 medium (g/L: 4.0 glucose; 4.0 yeast extract; 10.0 malt extract; distilled water; Oxoid, Basingstoke, UK) grown at 150 rpm and 28 °C, 2 mL were transferred to each 1000 mL Erlenmeyer flask containing 200 mL of ISP2 (pH adjusted to 7.0 ± 0.2). The 12 flasks were incubated in dark conditions at 28 °C and 150 rpm for 13 days. The fermentation was then filtered with sterile gauze, and the filtrate was subjected to liquid–liquid extraction (LLE). Briefly, the 2.4 L of culture was divided into 150 mL volumes that were extracted twice with ethyl acetate (1:3 *v*/*v*) (analytical grade, Synth^®^) in a separation funnel [55]. Finally, the organic phase was concentrated at 35 °C in a rotary evaporator (R-215 Buchi, Flawil, Switzerland) under reduced pressure conditions (100 mbar). The dry residues obtained were dissolved in methanol and filtered using 0.22 µm PTFE membrane filters (Merck, Cork, Ireland). Antimicrobial activity tests, ultra-performance liquid chromatography coupled with mass spectrometry (UPLC-MS) analysis, and purification of active compounds by preparative high-performance liquid chromatography (Prep-HPLC) were performed on the crude extract. The uninoculated ISP2 medium was extracted with ethyl acetate and tested with the target strains to rule out any antimicrobial effect.

#### 4.4.2. UPLC-MS Analysis

The Caat 5-35 fractions were analyzed in an Acquity UPLC^®^ system coupled to a Xevo TQ-S triple quadrupole mass spectrometer (Waters Corporation, Milford, MA, USA), equipped with an Acquity H Class quaternary pump and electrospray ionization (ESI) source. For analysis, 4 µL of the methanolic fraction was injected into an Ascentis^®^ Express C18 column (3.3 × 100 mm, 2.7 µm; Sigma-Aldrich, Bellefonte, PA, USA) operating at 35 °C and a flow rate of 0.4 mL/min. The mobile phase (A) consisted of 0.1% formic acid in water, while phase (B) consisted of 0.1% formic acid in acetonitrile (ACN) (Merck, Darmstadt, Germany). The elution gradient programme was as follows: 0.0–8.0 min, 30–95% B; 8.0–12.0 min, 95–98% B; 12.1–15.0 min, 30–30% B. The electrospray ionization (ESI) source operated in both positive and negative modes, and the mass spectrometer was run under previously reported conditions [56], with the following modifications: capillary voltage set to 40 V, desolvation gas flow at 700 L/h, and nebulizer gas pressure at 6 bar. The spectra were analyzed using MassLynx V4.1 SCN 803 software (Waters^®^, Milford, MA, USA).

#### 4.4.3. Preparative HPLC Fractionation

The Caat-535 culture extract, dissolved in methanol, was injected into a Shim-pack PREP-ODS (H) reverse-phase column (20 × 250 mm, 5 µm, Shimadzu, Kyoto, Japan) in an HPLC system (LC-6AD, Shimadzu, Kyoto, Japan) equipped with a DGU-20A5 degasser and CBM-20A controller. The solvent used for the separation was HPLC-grade (Merck). The antimicrobial extracts were fractionated at 15 mL/min using water (A) and ACN (B) as mobile phases, both containing 0.1% formic acid. The gradient elution strategy consisted of the following programme: 0.0–25.0 min, from 30 to 98% B; 25.0–50.0 min, from 98 to 98% B. The fractions were collected with the FRC-10A collector (Shimadzu, Kyoto, Japan), and absorbance was monitored at 325 and 370 nm with an SPD-20A detector. All fractions were evaporated, and the dried residues were filtered and used for bioassays with phytopathogenic microorganisms and mass spectrometry analyses.

### 4.5. In Vitro Antimicrobial Activity Bioassays

The effect of Caat 5-35 extracts and fractions against *P. palmivora* APB-35 was evaluated using the disc diffusion method in carrot agar [57,58]. For the assay, an agar plug containing oomycete mycelium (from a 7-day-old culture) was placed in the centre of a Petri dish, and sterile discs impregnated with 100 µg/mL of the extract or fraction were placed at four equidistant sites. The control plate contained only the same volume of the solvent used in the treatments, methanol or dimethyl sulfoxide (DMSO) (Merck, Darmstadt, Germany), in addition to the pathogen. The bioassays were incubated in the dark at 30 °C until the control mycelium reached the edge of the Petri dish. Anti-oomycete activity was determined by mycelial growth inhibition around the disc. The tests were carried out in three independent replicates.

Antifungal activity against *F. oxysporum*, *C. gloeosporioides*, and *R. solani* was evaluated using the disc diffusion method [57]. In fungal bioassays, a suspension of conidia (10^6^ spores/mL) or mycelium for *R. solani* (10^6^ CFU/mL) was spread over the surface of PDA medium. Then, sterile paper discs, previously impregnated and evaporated with 100 µg/mL of the extract or methanolic fraction, were placed on top. The plates were incubated in dark conditions at 30 °C for 48–72 h. Discs with evaporated methanol and difenoconazole (FUNGIWAY^®^, Goiás, Brazil) were used as negative and positive controls, respectively. Antifungal activity was determined by measuring the inhibition halo around the disc. All experiments were performed in triplicate.

Bioassays of extracts or fractions against the phytopathogenic bacteria *P. carotovorum*, *R. solanacearum* EAP05, and *Xcc*, unlike the previous one, were performed in AKM and using a suspension of 10^8^ CFU/mL of the phytopathogens as previously described [56,57]. The Petri dishes were incubated at 30 °C for 24–48 h in dark conditions. Tetracycline (100 µg/mL, Sigma, St. Louis, MO, USA) was used as a positive control, and a disk with evaporated methanol as a negative control. Antibacterial activity was determined by measuring the inhibition halo around the disc. All experiments were performed in triplicate. Samples that exhibited antimicrobial activity were analyzed by using UPLC-MS, UHPLC-triple-TOF-MS/MS, or nuclear magnetic resonance (NMR).

The activity index (AI) against *P. carotovorum* and *C. gloeosporioides* was calculated using the average inhibition halos from the disk diffusion assays of fractions 2, 3, 4, and 11 and the positive control. Where AI = (inhibition zone of the compound/inhibition zone of the positive control) × 100% [59].

### 4.6. Identification of Antimicrobial Metabolites by UHPLC-TOF-MS/MS and Nuclear Magnetic Resonance (NMR) Analysis

The extract and fractions exhibiting antimicrobial activity were analyzed using the ultra-high performance liquid chromatography (UHPLC) system (Nexera X2, Shimadzu, Kyoto, Japan) equipped with an LC-30AD binary pump, DGU-20A_5R_ degasser, SIL-30AC autosampler, CTO-30A column oven, and a CBM-20A controller. The system was coupled to a TripleTOF™ 5600+ mass spectrometer (AB Sciex, Foster City, CA, USA) equipped with a DuoSpray™ ion source, operated under the same chromatographic column, gradient, and acquisition conditions, as well as MS and MS/MS data analysis parameters previously established by our group [56].

A total of 8.5 mg of fraction 13, dissolved in deuterated chloroform (Sigma-Aldrich), was analyzed in a DRX 500 spectrometer (Bruker^®^, Billerica, MA, USA) at frequencies of 500 MHz for (^1^H) and 125 MHz for (^13^C). The spectra obtained were processed using ACD/NMR Processor Academic Edition version 12.01 (ACD/Labs) and MestReNova version 6.0.2 (Mestrelab Research) software.

### 4.7. Detection of Cellulases and Phosphate Solubilization by Streptomyces *sp.* Caat 5-35

The ability of Caat 5-35 to hydrolyze cellulose was evaluated using a culture medium with carboxymethylcellulose (CMC) as the sole carbon source, with the following composition g/L: 1.0 K_2_HPO_4_; 1.0 KH_2_PO_4_; 0.2 MgSO_4_ 7H_2_O; 1.0 NH_4_NO_3_; 0.05 FeCl_3_ 6H_2_O; 0.02 CaCl_2_; 1% CMC and 15% agar [60]. All the salts used in the study were purchased from Merck (Darmstadt, Germany) and Sigma (St. Louis, MO, USA). 20 µL of a Caat 5-35 bacterial suspension (10^8^ CFU/mL) was inoculated into five mm-diameter wells of CMC agar. After 5 days of incubation at 28 °C, the solid medium was flooded with a 0.05% Congo Red solution for 10 min. The surface was then washed several times with 1.0 M NaCl. Finally, the hydrolysis halo or clearing zone around the well was measured. All experiments were performed in triplicate.

A Caat 5-35 colony previously grown on ISP2 agar was seeded on NBRIP agar (g/L: 10 glucose; 5 Ca_3_(PO_4_)_2_; 5 MgCl_2_ 6H_2_O; 0.25 MgSO_4_ 7H_2_O; 0.2 KCl; 0.1 (NH_4_)_2_SO_4_; 15 agar, pH 7.0) [61]. After 15 days of incubation in dark conditions at 28 °C, the growth diameter of the bacteria and the solubilization halo were measured to determine the phosphate solubilization index (PSI). Where PSI = (colony diameter + halo diameter)/colony diameter.

### 4.8. In Vitro Test on Cacao Fruits

The biocontrol potential of strain Caat 5-35 was evaluated according to the protocol described previously with some modifications [43,62]. Briefly, the surface of 4- to 5-month-old cacao pods was disinfected for 2 min with 70% ethanol, washed three times with sterile water, and dried at room temperature. Then, using sterile 1 mL tips, a circular wound 5 mm in diameter and depth was made on each pod, followed by a puncture in the centre of the wound. Twenty µL of the bacterial suspension (10^8^ CFU/mL) of Caat 5-35 was inoculated into this area. The cacao fruits were placed in a humid chamber fitted with plastic trays, absorbent paper, inverted Petri dishes, and distilled water, all of which were autoclaved previously. Incubation was carried out for 24 h to promote bacterial colonization. After this period, each wound was inoculated with an agar plug (5 mm in diameter) containing *P. palmivora* APB-35 mycelium. Twenty-four and 48 h after pathogen inoculation, 20 µL of the Caat 5-35 suspension was reapplied to the same site. The pods were kept in a humid chamber for 6 days at 28 °C under a 12 h light:12 h dark cycle. After the incubation period ended, the lesion area was calculated as described in the literature [63]. The experiment consisted of five biological replicates per treatment and was independently repeated, resulting in a total of n = 10. Cacao fruits treated with sterile water without a pathogen or inoculated with the oomycete served as controls.

### 4.9. Statistical Analysis

A completely randomized single-factor design was used in this study. A one-way analysis of variance (ANOVA) was performed on the data obtained. Statistical differences were evaluated using Tukey’s test with a 95% confidence level (α = 0.05). Means were considered significantly different if the *p*-value was ≤0.05. Data were analyzed using Minitab^®^ version 17 (Minitab, State College, PA, USA).

## 5. Conclusions

The actinomycete *Streptomyces* sp. Caat 5-35, isolated from the soil of the Caatinga biome in Brazil, shows an antagonistic effect against the phytopathogens *P. palmivora* APB-35, *C. acutatum*, *S. sclerotiorum*, *F. oxysporum*, *F. graminearum*, and *R. solani*. According to the available evidence, this is the first study to demonstrate the antifungal activity of albofungin, its derivatives, and albonoursin against *P. palmivora*. Additionally, the inhibition of *C. gloeosporioides* growth by albonoursin, as well as the antibacterial effect of 2-chloroadenosine and 5′-O-sulfamoyl-2-chloroadenosine against *P. carotovorum*, are novel findings. However, for a comprehensive evaluation of the potential of Caat 5-35, further dose–response studies are needed, including determinations of minimum inhibitory concentration (MIC) and IC50, as well as *in vivo* bioassays with purified compounds, extract, and cell-free supernatant. The results obtained in the *in vitro* assay suggest that *Streptomyces* sp. Caat 5-35 may have potential use for the biological control of diseases caused by *P. palmivora*, *C. gloeosporioides*, and *P. carotovorum*.

## Figures and Tables

**Table 1:** Inhibition halo produced by fractions and the extract of *Streptomyces* sp. Caat 5-35 against the phytopathogenic strain. The intervals represent the standard error of the mean (n = 3). Means in the same column that do not share a letter are significantly different according to Tukey’s test (α = 0.05). The extract and fractions were evaluated at a final concentration of 100 µg/mL. ND: not determined. Positive control: Difenoconazole (FUNGIWAY^®^). Negative control: disc with evaporated methanol. Disk size: 5.0 mm.

Treatment	Inhibition Halo of Phytopathogens (mm)	
	*P. palmivora*	*C. gloeosporioides*
Extract	16.0 ± 1 bc	27.0 ± 1 ab
Fraction 8	10.0 ± 1 e	15.0 ± 1 e
Fraction 10	17.5 ± 0.5 ab	20.0 ± 2.6 de
Fraction 11	17.7 ± 0.8 ab	25.7 ± 1.5 b
Fraction 12	14.0 ± 0.5 cd	27.3 ± 2.1 ab
Fraction 13	19.0 ± 1 a	27.5 ± 1.8 ab
Fraction 14	17.5 ± 0.5 ab	28.0 ± 2 ab
Fraction 15	15.0 ± 0.5 c	25.3 ± 1.2 bc
Fraction 16	12.5 ± 0.5 d	17.0 ± 2.6 de
Fraction 17	10.0 ± 1 e	17.0 ± 1 de
Fraction 18	10.0 ± 1 e	20.3 ± 2.1 cd
Difenoconazole	ND	31.0 ± 1 a
Negative Control	0.0 ± 0.0 f	0.0 ± 0.0 f

**Table 2:** Antimicrobial activity of chlorinated ribonucleosides and albonoursin produced by *Streptomyces* Caat 5-35 against phytopathogenic strain. The intervals represent the standard error of the mean (n = 3). Means in the same column that do not share a letter are significantly different according to Tukey’s test (α = 0.05). The extract and fractions were evaluated at a final concentration of 100 µg/mL. Positive control: tetracycline *, Difenoconazole **. Negative control: disc with evaporated methanol. F^2^: Fraction 2. F^3^: Fraction 3. F^4^: Fraction 4. Disk size: 5.0 mm.

Treatment	Phytopathogens					
	*Pectobacterium carotovorum*			*Colletotrichum gloeosporioides*		
	Inhibition Zone of Compound (mm)	Inhibition Zone of Positive Control (mm) *	Activity Index (%)	Inhibition Zone of Compound (mm)	Inhibition Zone of Positive Control (mm) **	Activity Index (%)
Extract	19.0 ± 1 b	39 ± 1	48.7	27.0 ± 1 a	31.0 ± 1	87.1
2-chloroadenosine and 5′-O-sulfamoyl-2-chloroadenosine	27.7 ± 2.5 a ^F2^	39 ± 1	71	0 ± 0 b	31.0 ± 1	0
	27.3 ± 2.5 a ^F3^	39 ± 1	70	0 ± 0 b	31.0 ± 1	0
	31.3 ± 1.2 a ^F4^	39 ± 1	80.2	0 ± 0 b	31.0 ± 1	0
Albonoursin	0 ± 0 c	39 ± 1	0	25.7 ± 1.5 a	31.0 ± 1	82.9
Negative control	0 ± 0 c	39 ± 1	0	0 ± 0 b	31.0 ± 1	0

**Table 3:** Compound assignment to experimental masses obtained by HRMS (ESI-TOF) of fractions with anti-oomycete activity against *P. palmivora* APB-35. ND: not determined.

Anti-Oomicete Activity	Fraction	[M+H]^+^	[M+Na]^+^	[2M+H]^+^	Assignment
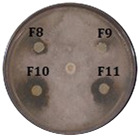	10	478.1498	500.1313	955.2938	Chrestoxanthone C
		507.1413	529.1222	1013.2768	Albofungin A
	11	257.1293	279.1110	513.2513	Albonoursin
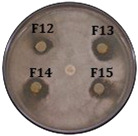	12	257.1294	279.1110	513.2514	Albonoursin
		291.1132	313.0957	581.2189	Piperafizine B
		506.1458	528.1272	1011.2852	Chrestoxanthone A
	13	521.1568	543.1382	1041.3073	Albofungin
	14	521.1568	543.1382	1041.3073	Albofungin
		520.1621	542.1434	1039.3161	Albofungin B
	15	521.1575	543.1392	1041.3090	Albofungin
		520.1621	542.1434	1039.3161	Albofungin B
		477.1547	499.1369		ND
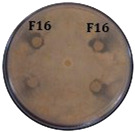	16	555.1169	577.0990	1109.2281	Chloroalbofungin

## Data Availability

The original contributions presented in this study are included in the article/Supplementary Materials. The 16S rDNA sequence of strain Caat 5-35 was deposited in the NCBI GenBank database (https://www.ncbi.nlm.nih.gov/genbank/ submitted on 20 September 2025) under accession number PX368896. Further inquiries can be directed at the corresponding author.

## Supplementary Materials

The following supporting information can be downloaded at: https://www.mdpi.com/article/10.3390/molecules31010021/s1, Figure S1: Neighbour-Joining (NJ) phylogenetic tree constructed from the 16S rRNA sequence of *Streptomyces* sp. Caat 5-35; Figure S2: Maximum Likelihood (ML) phylogenetic tree of *Streptomyces* sp. Caat 5-35 constructed based on the 16S rRNA; Figure S3: UHPLC-MS spectra of active compounds derived from Albofungin, [M+H]^+^ ions of *m*/*z* 478.1498 and 507.1413; Figure S4: UHPLC-MS spectra of active compounds derived from albofungin, [M+H]^+^ ions of *m*/*z* 506.1458 and 520.1621; Figure S5: UHPLC-MS spectrum of active compound derived from albofungin, ion [M+H]^+^ with *m*/*z* 555.1169; Figure S6: UHPLC-MS spectrum in positive mode of albofungin; Table S1: 1D and 2D NMR data for fraction 13, correlations and values reported in the literature for albofungin; Figure S7: 1H NMR spectrum of fraction 13 obtained from the culture of *Streptomyces* sp. Caat 5-35 in ISP2 medium; Figure S8: Spectrum of gHMQC (Heteronuclear Multiple Quantum Coherence) and gHMBC (Heteronuclear Multiple Bond Coherence) of fraction 13 obtained from the culture of *Streptomyces* sp. Caat 5-35 in ISP2 medium.
