# Supplementary material for: Novel Antimicrobial Activities of Albofungin, Albonoursin, and Ribonucleosides Produced by Streptomyces sp. Caat 5-35 Against Phytopathogens and Their Potential as a Biocontrol Agent
Source: Molecules. 2025 Dec 20;31(1):21. doi: 10.3390/molecules31010021 (PMC12786410; doi:10.3390/molecules31010021)
Supplement: Supplementary file 1 [file molecules-31-00021-s001.zip › molecules-4024416-Supplementary.pdf]

molecules

MDPI

Supplementary material

Novel Antimicrobial Activities of Albofungin, Albonoursin, and Ribonucleosides Produced by *Streptomyces* sp. Caat 5-35 Against Phytopathogens and Their Potential as a Biocontrol Agent

Carmen Julia Pedroza-Padilla [1,*], Sergio Orduz [2,*], Danilo Tosta Souza [3,4], Geraldo Astolpho Barbão [3] and Luiz Alberto Beraldo Moraes [3,*]

[1] Grupo BiotecGen, Departamento de Biología, Microbiología y afines, Facultad de Ciencias Básicas, Universidad Popular del Cesar, Diagonal 21 # 29-56, 200004, Valledupar, Colombia; carmenpedroza@unicesar.edu.co
[2] Departamento de Biociencias, Facultad de Ciencias, Universidad Nacional de Colombia, sede Medellín, Carrera 65 # 59A-110, 050034, Medellín, Colombia; sorduzp@unal.edu.co
[3] Departamento de Química, Faculdade de Filosofia, Ciências e Letras de Ribeirão Preto-FFCLRP, Universidade de São Paulo-USP, Ribeirão Preto, 14040-901, SP, Brazil; luizmoraes@ffclrp.usp.br
[4] Embrapa Meio Ambiente, Jaguariúna, São Paulo, 13918-110, SP, Brazil; danilo_tosta@hotmail.com

* Correspondence: carmenpedroza@unicesar.edu.co

ORCID: CJPP: 0000-0003-2233-0898; **SO**: 0000-0001-7587-3816; **DTS**: 0000-0002-8093-6663 and **LABM**: 0000-0002-0853-1944.

FIGURES AND SUPPLEMENTARY TABLE

Figure S1. Neighbor-Joining (NJ) phylogenetic tree constructed from the 16S rRNA sequence of *Streptomyces sp.* Caat 5-35. The scale bar corresponds to 0.0050 nucleotide substitutions per site. *Streptomyces albus* subsp. *albus* DSM 40313 was used as an outgroup.
Figure S2. Maximum Likelihood (ML) phylogenetic tree of *Streptomyces sp.* Caat 5-35 constructed based on the 16S rRNA. The scale bar corresponds to 0.01 nucleotide substitutions per site. *Streptomyces albus* subsp. *albus* DSM 40313 was used as an outgroup.
Figure S3. UHPLC-MS spectra of active compounds derived from Albofungin, $[M+H]^+$ ions of *m/z* 478.1498 and 507.1413. Top: Chrestoxanthone C. Bottom: Albofungin A. Chemical structures illustrated using ChemDraw 21.0 software.
Figure S4. UHPLC-MS spectra of active compounds derived from albofungin, $[M+H]^+$ ions of *m/z* 506.1458 and 520.1621. Top: Chrestoxanthone A. Bottom: Albofungin B. Chemical structures illustrated using ChemDraw 21.0 software.
Figure S5. UHPLC-MS spectrum of active compound derived from albofungin, ion $[M+H]^+$ with *m/z* 555.1169. Chloroalbofungin. Chemical structure illustrated using ChemDraw 21.0 software.
Figure S6. UHPLC-MS spectrum in positive mode of albofungin. Amplified spectrum of the $[M+H]^+$ ion of *m/z* 521.1568 and chemical structure illustrated with ChemDraw 21.0 software.
Table S1. 1D and 2D NMR data for fraction 13, correlations and values reported in the literature for albofungin.
Figure S7. 1H NMR spectrum of fraction 13 obtained from the culture of *Streptomyces sp.* Caat 5-35 in ISP2 medium.

Figure S8. Spectrum of gHMQC (Heteronuclear Multiple Quantum Coherence) and gHMBC (Heteronuclear Multiple Bond Coherence) of fraction 13 obtained from the culture of *Streptomyces sp.* Caat 5-35 in ISP2 medium.

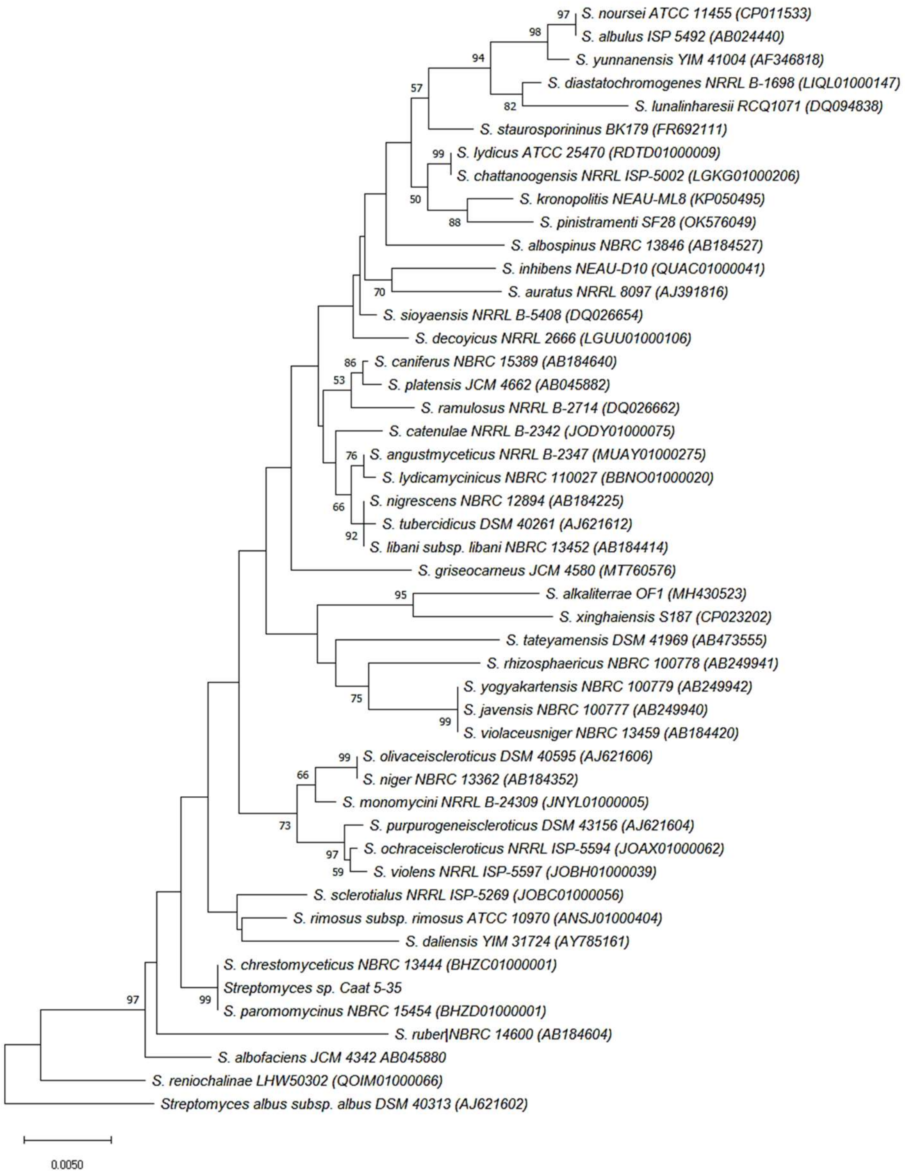

Figure S1. Neighbor-Joining (NJ) phylogenetic tree constructed from the 16S rRNA sequence of *Streptomyces sp.* Caat 5-35. The scale bar corresponds to 0.0050 nucleotide substitutions per site. *Streptomyces albus* subsp. *albus* DSM 40313 was used as an outgroup.

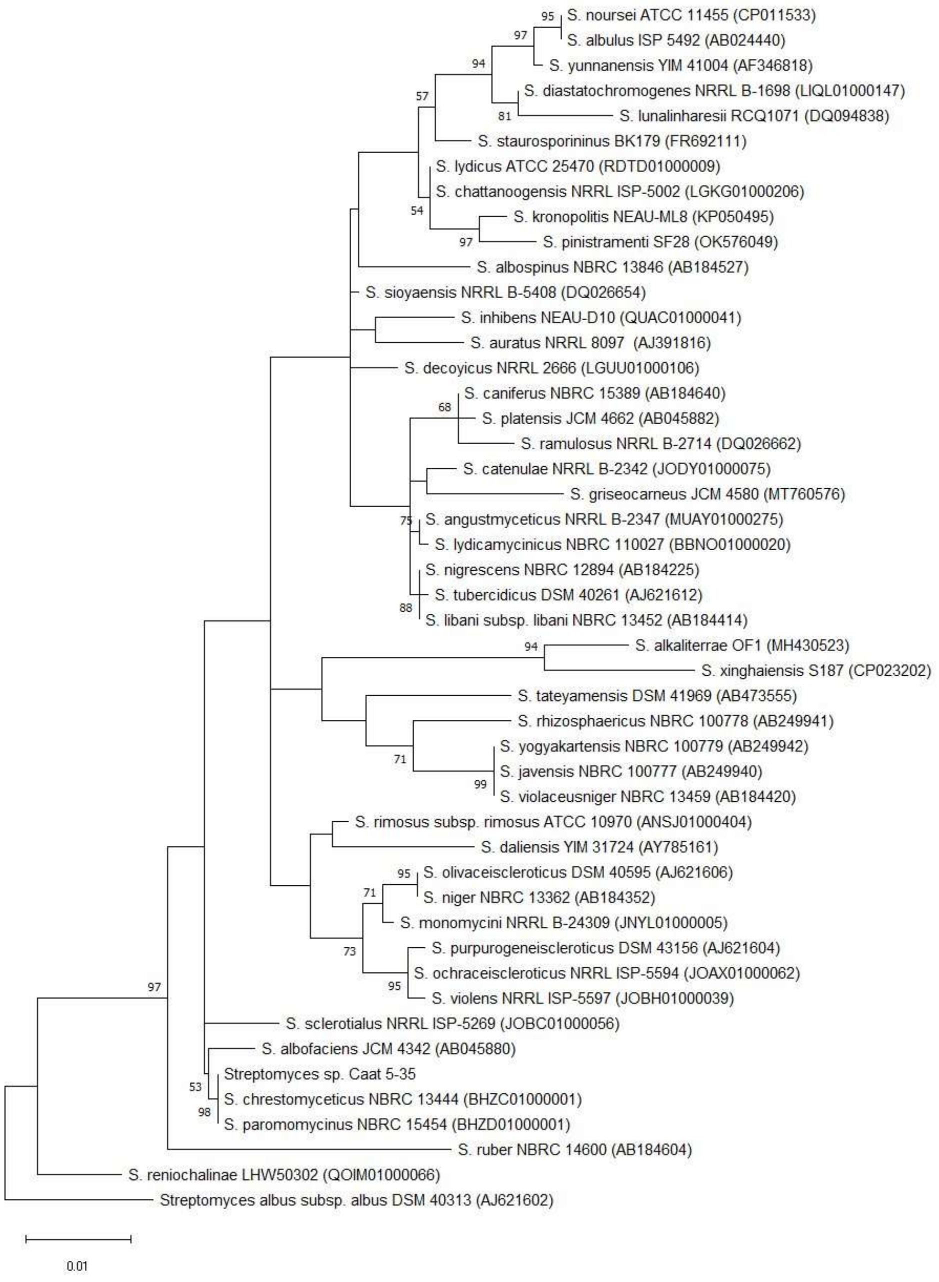

Figure S2. Maximum Likelihood (ML) phylogenetic tree of *Streptomyces sp.* Caat 5-35 constructed based on the 16S rRNA. The scale bar corresponds to 0.01 nucleotide substitutions per site. *Streptomyces albus* subsp. *albus* DSM 40313 was used as an outgroup.

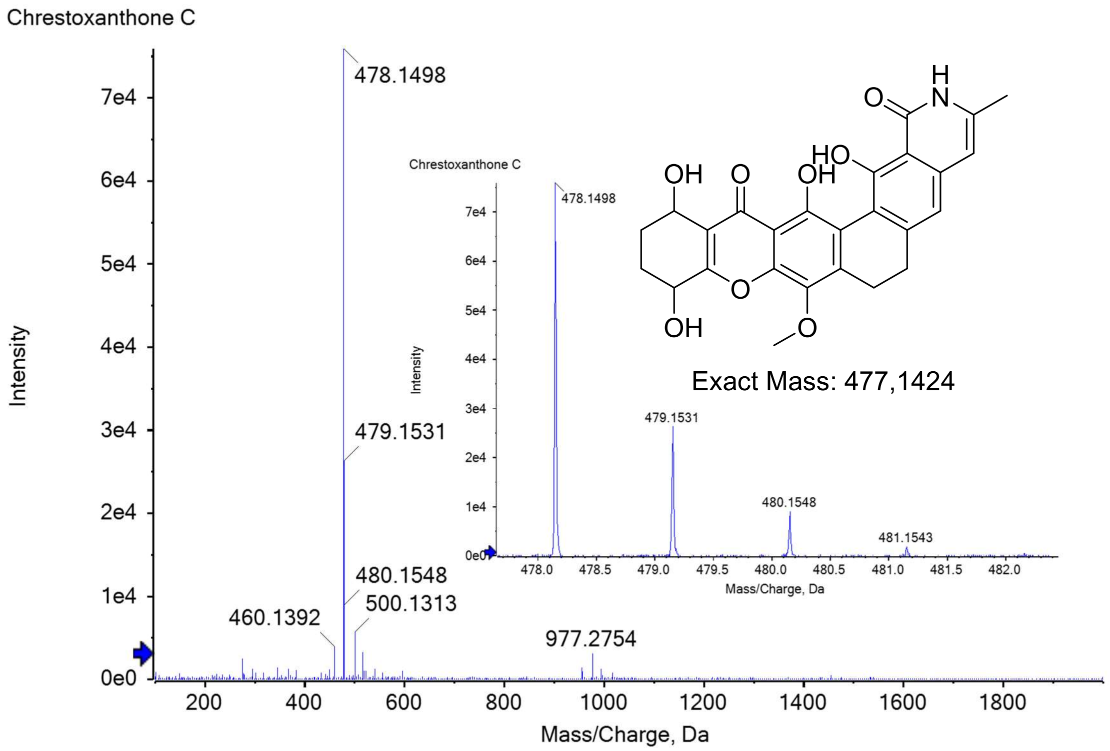

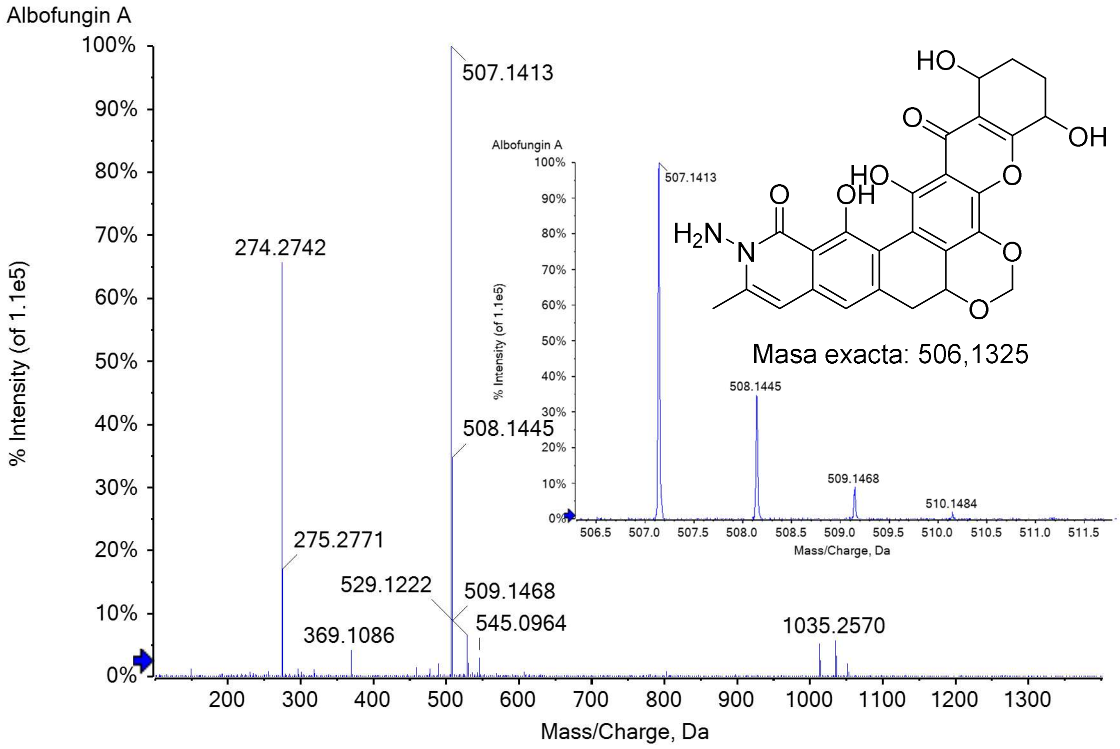

Figure S3. UHPLC-MS spectra of active compounds derived from albofungin, $[M+H]^+$ ions of *m*/*z* 478.1498 and 507.1413. Top: Chrestoxanthone C. Bottom: Albofungin A. Chemical structures illustrated using ChemDraw 21.0 software.

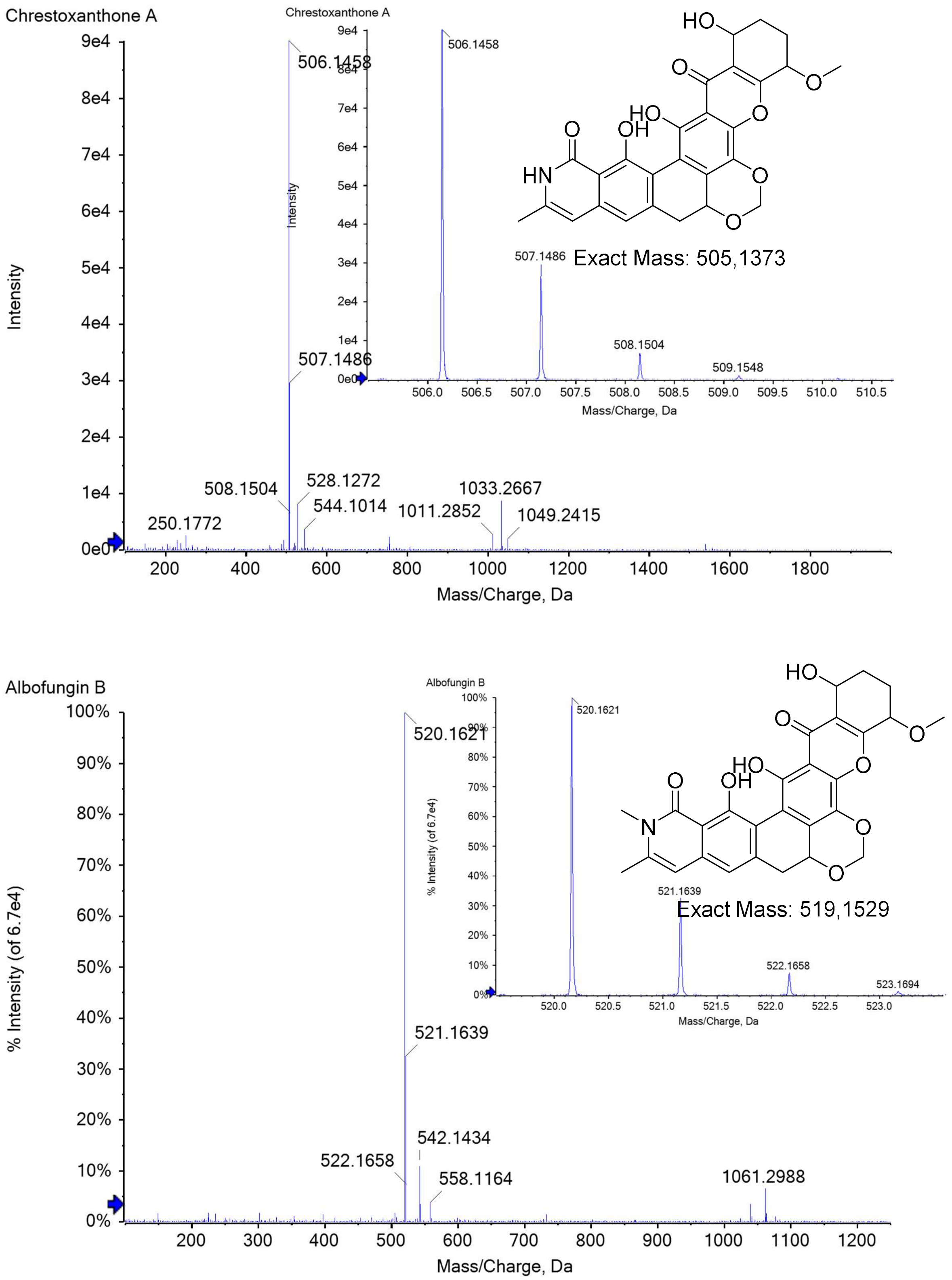

Figure S4. UHPLC-MS spectra of active compounds derived from albofungin, $[M+H]^+$ ions of *m*/*z* 506.1458 and 520.1621. Top: Chrestoxanthone A. Bottom: Albofungin B. Chemical structures illustrated using ChemDraw 21.0 software.

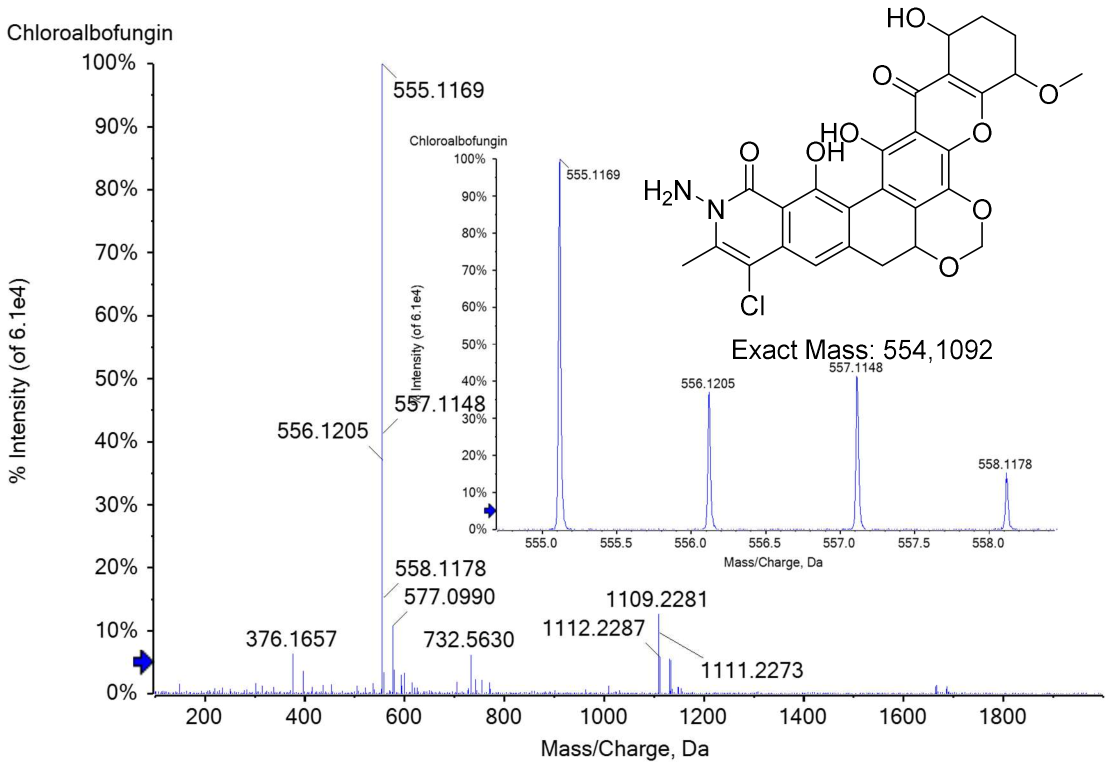

Figure S5. UHPLC-MS spectrum of active compound derived from Albofungin, ion [M+H]$^{+}$ with *m*/*z* 555.1169. Chloroalbofungin. Chemical structure illustrated using ChemDraw 21.0 software.

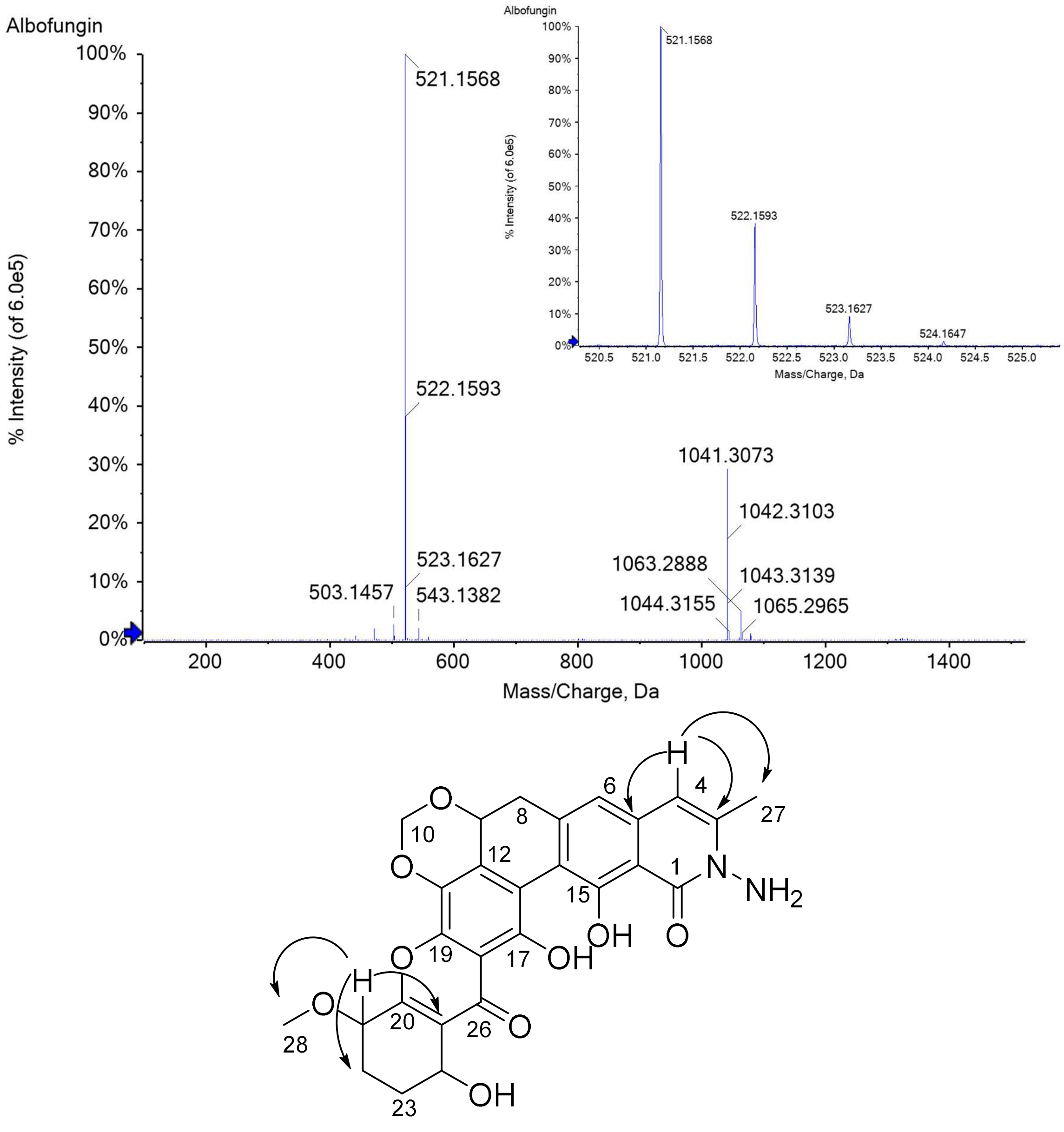

Figure S6. UHPLC-MS spectrum in positive mode of Albofungin. Amplified spectrum of the $[M+H]^+$ ion of *m*/*z* 521.1568 and chemical structure illustrated with ChemDraw 21.0 software.

molecules

MDPI

Table S1. 1D and 2D NMR data for fraction 13 correlations and values reported in the literature for Albofungin. F13: Albofungin of *Streptomyces sp.* Caat 5-35.

**Position**		**δ ^{13}C (ppm)**		**δ ^{1}H (ppm), m (J,Hz)**		**HMBC correlation (ppm)**
**F13**	**a***	**F13**	**a***	**F13**	**a***	**F13**
1	1	171.3	163.3			
2	2	-	-			
3	3	140.9	141.7			
4	4	106.0	105.2	6.37, s	6.61 (1H, s)	140.9; 114.3; 110.3; 19.6
5	5	110.3	136.3			
6	6	113.9	113.8	6.84, s	7 (1H, s)	37.0; 114.3; 110.3; 106.0
7	7	130.3	140.4			
8	8	37.0	35.9	3.18, dd (4.6; 13.7) 2.91, dd (4.6; 13.7)	3.22 (2H, dd, 13.8, 4.4) 2.76 (2H, t 13)	72.8; 113.5; 130.3; 140.9
9	9	72.9	72.1	4.98	4.96 (1H, dd, 12.9, 4.6)	37.0
10	10	90.8	90.6	5.33, d (5.9) 5.56, d (5.9)	5.42 (2H, d, 6.1) 5.61 (2H, d, 6.1)	72.9; 130.3
11	11	130.3	130.3			
12	12	140.9	130.1			
13	13	112.7	109.8			

14	14	113.5	112.8			
15	15	157.6	156.8			
16	16	109.8	109.2			
17	17	150.3	149.6			
18	18	110.1	111.6			
19	19	-	142.7			
20	20	164.2	165.1			
21	21	74.2	74.7	4.24, t (4.1)	4.42 (1H, t, 7.8)	58.5; 25.7; 120.5; 164.2
22	22	25.7	22.8	2.24 m<br>2.05, s	2.06 (2H, m)	74.2; 63.5; 164.2
23	23	24.4	27.9	1.89, m<br>2.05, s	1.72 (2H, m), 1.82 (2H, m)	
24	24	63.5	58.8	5.00, t (6.75)	4.82 (1H, d, 3.5), 5.14 (OH, d, 4.2)	163.0; 119.1; 25.7
25	25	119.1	120.3			
26	26	191.8	182.1			
27	27	19.3	18.9	2.51, s	2.45 (3H, s)	106.0; 140.9
28	28	58.5	57.8	3.64, s	3.57 (3H, s)	73.1
OH				2.72, s		191.4
OH				12.66, s	12.95 (OH,s)	110.1; 112.7; 150.3

OH				13.40, s	13.58 (OH,s)	109.8; 157.6; 113.5
NH_2				2.05, s		171.3

F13: purified fraction 13 of albofungin from *Streptomyces sp.* Caat 5-35 extract. **a***: reported albofungin by Wu et al., 2018. **b***. s: singlet; d: dublet; m: no defined multiplicity.

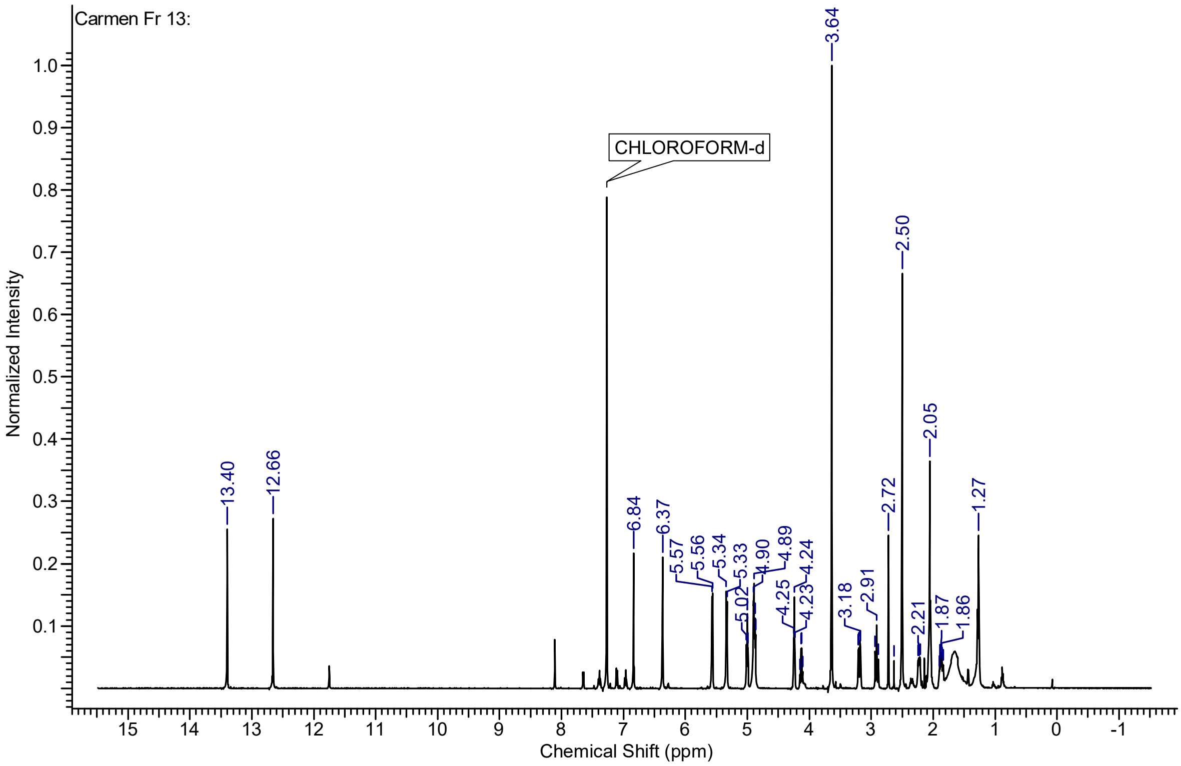

Figure S7. 1H NMR spectrum of fraction 13 obtained from the culture of *Streptomyces sp.* Caat 5-35 in ISP2 medium.

Figure S8. Spectrum of gHMQC (Heteronuclear Multiple Quantum Coherence) and gHMBC (Heteronuclear Multiple Bond Coherence) of fraction 13 obtained from the culture of *Streptomyces sp.* Caat 5-35 in ISP2 medium (**below**).

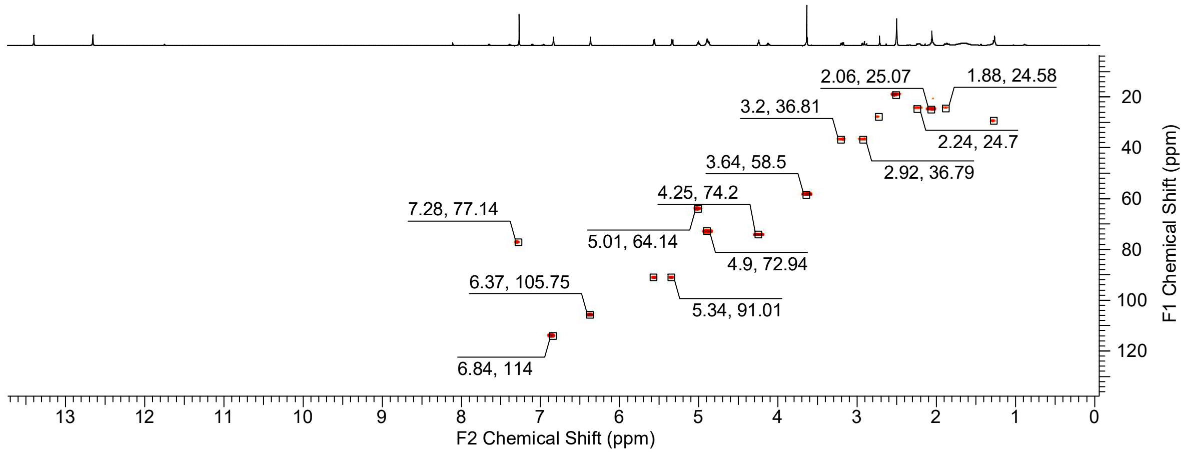

 https://doi.org/10.3390/molecules31010021

HMQC

Image of the magnified spectrum

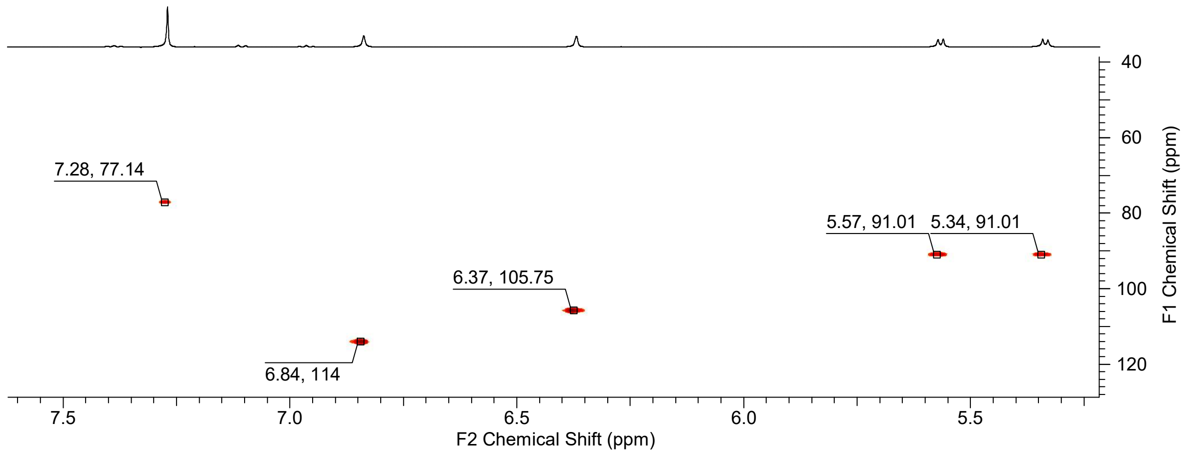

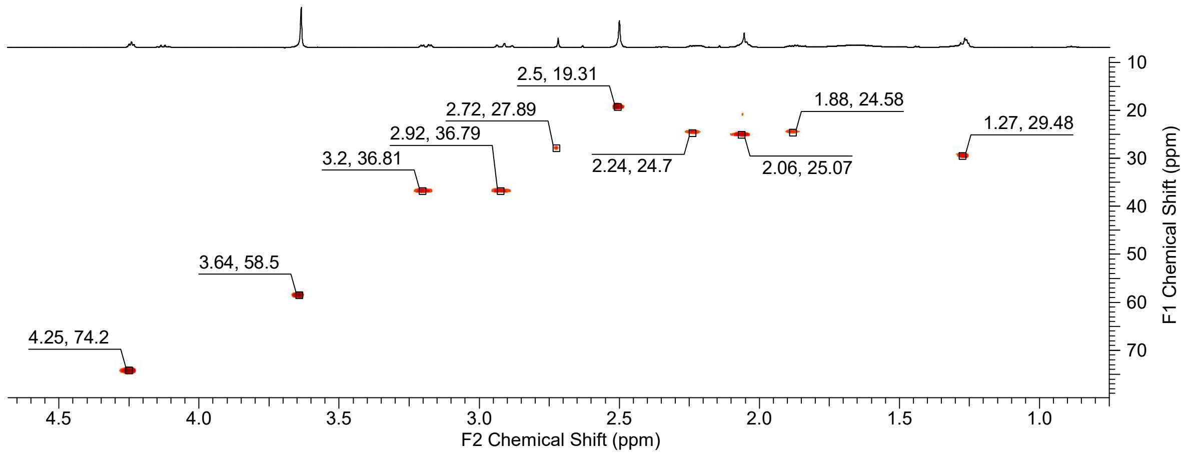

HMBC

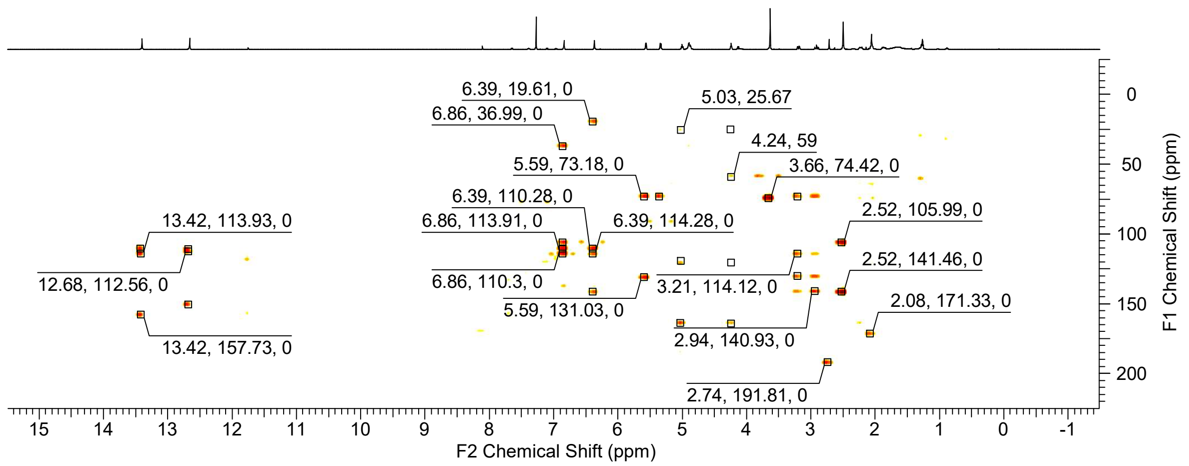

Image of the magnified spectrum

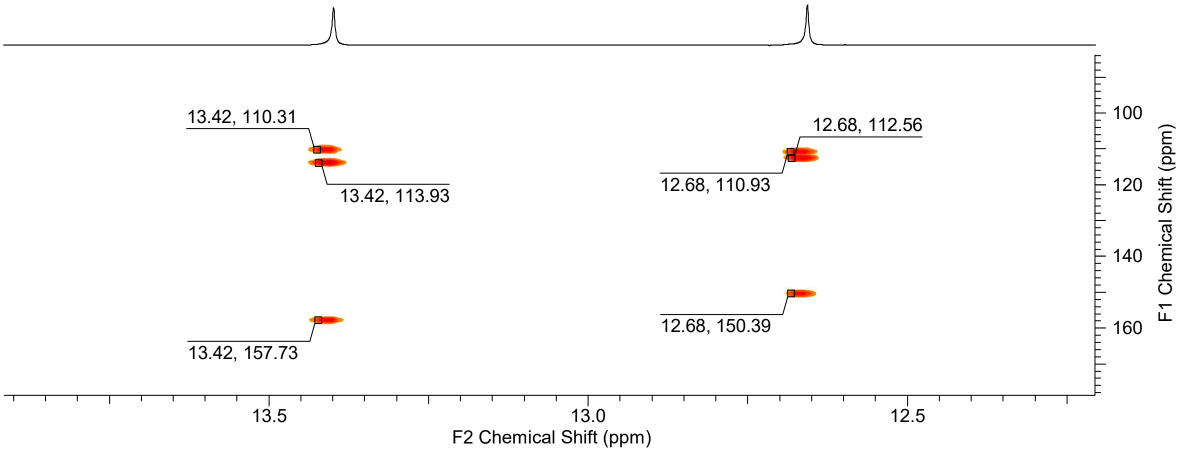

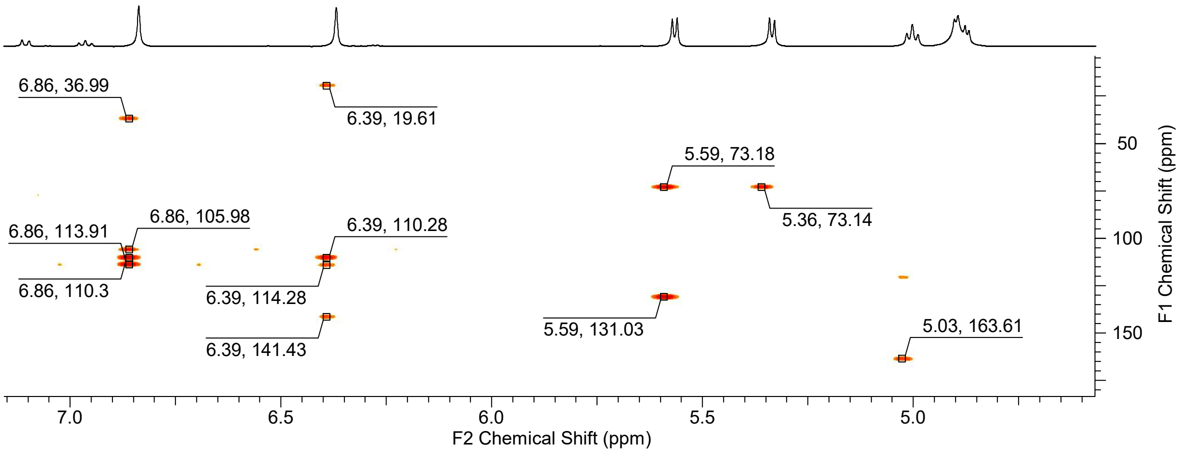

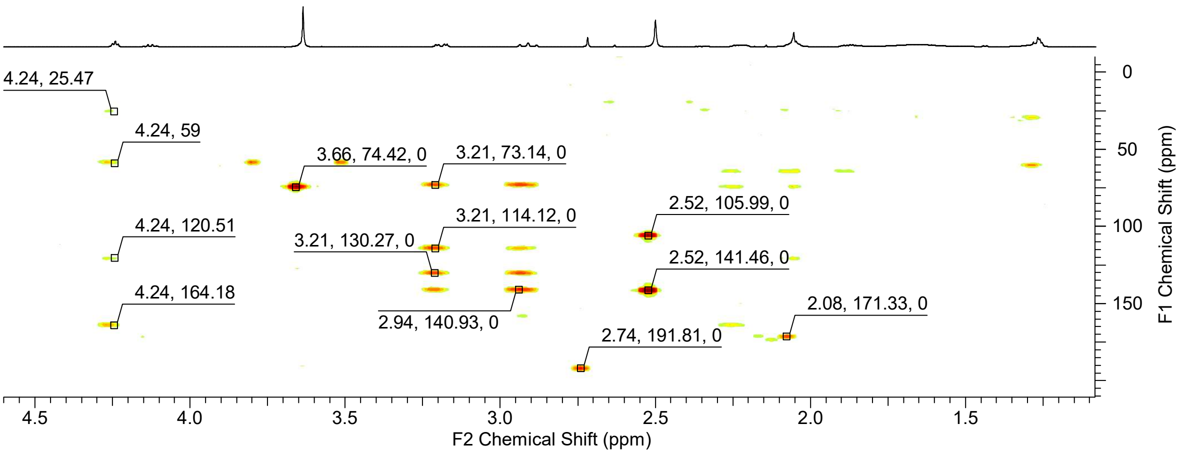

Disclaimer/Publisher's Note: The statements, opinions and data contained in all publications are solely those of the individual author(s) and contributor(s) and not of MDPI and/or the editor(s). MDPI and/or the editor(s) disclaim responsibility for any injury to people or property resulting from any ideas, methods, instructions or products referred to in the content.
